# Greater fuel efficiency is potentially preferable to reducing NO_*x*_ emissions for aviation’s climate impacts

**DOI:** 10.1038/s41467-020-20771-3

**Published:** 2021-01-25

**Authors:** Agnieszka Skowron, David S. Lee, Rubén Rodríguez De León, Ling L. Lim, Bethan Owen

**Affiliations:** Faculty of Science and Engineering, Manchester Metropolitan University, Manchester, UK

**Keywords:** Atmospheric chemistry, Climate-change impacts, Climate-change mitigation

## Abstract

Aviation emissions of nitrogen oxides (NO_*x*_) alter the composition of the atmosphere, perturbing the greenhouse gases ozone and methane, resulting in positive and negative radiative forcing effects, respectively. In 1981, the International Civil Aviation Organization adopted a first certification standard for the regulation of aircraft engine NO_*x*_ emissions with subsequent increases in stringency in 1992, 1998, 2004 and 2010 to offset the growth of the environmental impact of air transport, the main motivation being to improve local air quality with the assumed co-benefit of reducing NO_*x*_ emissions at altitude and therefore their climate impacts. Increased stringency is an ongoing topic of discussion and more stringent standards are usually associated with their beneficial environmental impact. Here we show that this is not necessarily the right direction with respect to reducing the climate impacts of aviation (as opposed to local air quality impacts) because of the tradeoff effects between reducing NO_*x*_ emissions and increased fuel usage, along with a revised understanding of the radiative forcing effects of methane. Moreover, the predicted lower surface air pollution levels in the future will be beneficial for reducing the climate impact of aviation NO_*x*_ emissions. Thus, further efforts leading to greater fuel efficiency, and therefore lower CO_2_ emissions, may be preferable to reducing NO_*x*_ emissions in terms of aviation’s climate impacts.

## Abstract (web summary)

The regulation of aircraft engine NO_*x*_ emissions was introduced to improve local air quality and reduce NO_*x*_ emissions at altitude. Here, the authors find that greater fuel efficiency of aircrafts, and therefore lower CO_2_ emissions, could be preferable to reducing NO_*x*_ emissions in terms of the aviation industries future climate impacts.

## Introduction

Emission standards for aircraft NO_*x*_ are set by the Committee on Aviation Environmental Protection of the International Civil Aviation Organisation (ICAO-CAEP). In the past, aircraft NO_*x*_ emissions standards have been set to protect local air quality and have been assumed to have co-benefits for climate protection, as aircraft NO_*x*_ results in an overall warming effect at present^1,2^. Emissions of NO_*x*_, whether from aviation or other sources, result in the short-term formation of ozone (O_3_) (a warming) and the long-term destruction, via hydroxyl (OH) production, of small amounts (~a few percent) of ambient methane (CH_4_) (a cooling)^1^. In addition, the methane reduction results in a long-term reduction in O_3_ (cooling)^3^ and a long-term reduction in H_2_O in the stratosphere (cooling) from reduced oxidation of methane^4^. The net balance of these components ranges from positive for aviation NO_*x*_, to negative for shipping and surface NO_*x*_^4–6^.

The general scientific advice given to the ICAO-CAEP to date has been to reduce emissions of both NO_*x*_ and CO_2_. However, reducing both is problematic because of a technological trade-off between aviation NO_*x*_ and CO_2_^7–9^. Furthermore, one has to be very careful trading short-lived climate forcers against long-lived greenhouse gases, e.g. the reduction of NO_*x*_ emissions might result in a fuel penalty that in fact can lead to a net climate disbenefit^10^. Here, we present a new analysis of future aviation emission scenarios and the balance of NO_*x*_ from surface and aircraft sources, that makes this recommendation uncertain in terms of climate benefits and we suggest that the scientific evidence for reducing aircraft NO_*x*_ needs to be revisited.

At subsonic aircraft cruise altitudes of 8–12 km, the atmosphere is sensitive to aircraft NO_*x*_ emissions where O_3_ production is four times more efficient than near the ground^11^ and the aviation net NO_*x*_ effect also depends on the state of the atmosphere into which NO_*x*_ is emitted^12^. Changes in emissions of any surface source that take place as a result of various air quality and climate policies may have impacts on background conditions, and consequently might have an impact on the climate effect of aviation NO_*x*_ emissions, which is not independent of background conditions. The changes in the tropospheric composition and global radiative forcing (RF) for various scenarios of anthropogenic O_3_ precursor emissions have been widely explored^13–15^. However, the impact of changing surface emissions on aircraft NO_*x*_ climate effects has been virtually left out of discussions and sensitivity experiments can only be found in one study^3^. This present study aims to fill this gap and start the discussion on how future anthropogenic background emissions can affect the aviation climate impact.

Using a suitable three-dimensional chemistry transport model (CTM) of the global atmosphere (MOZART3)^16,17^, we examine the changes in the tropospheric composition and the net RF from aviation NO_*x*_ emissions for 30% reductions in the most recent present-day inventories available (2006) of O_3_ precursor emissions (NO_*x*_, carbon monoxide - CO, non-methane volatile organic compounds - NMVOC) and for a future (2050) range of Representative Concentration Pathways (RCP) scenarios together with ICAO-CAEP aviation emission projections (see Methods for details of models used and simulations). These simulations allow an analysis of the relative benefits to reducing aviation impacts from reducing aviation and background NO_*x*_ emissions.

## Results

### Aviation net NO_*x*_ radiative forcing in 2050

**Fig. 1:**
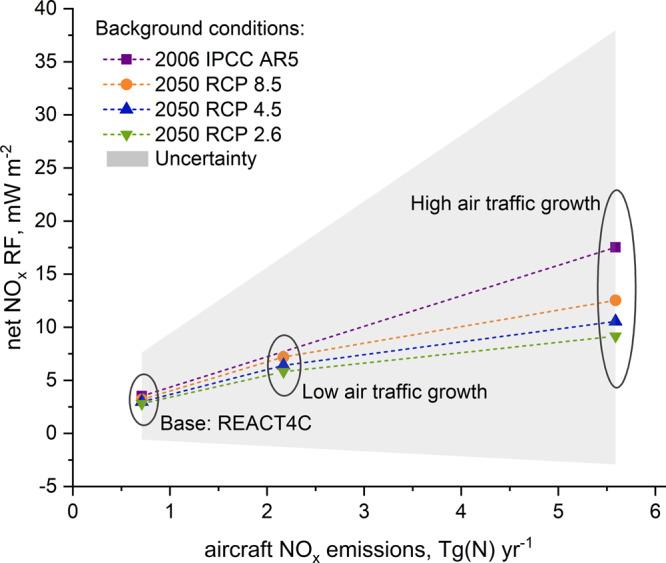
Net NO_*x*_ radiative forcing by emission rate, original CH_4_ parameterisation. Aviation net NO_*x*_ radiative forcing (RF) (the sum of the short-term positive O_3_ RF perturbation and the negative RF terms caused by a reduction in CH_4_ lifetime, see Methods), by aviation NO_*x*_ emission rate according to a range of background emission scenarios, utilising the IPCC Fifth Assessment Report^59^ simplified expression for the calculation of CH_4_ forcing. Aviation net NO_*x*_ RF systematically increases with increasing NO_*x*_ emissions from aviation, showing a variation according to the background surface emissions, with high mitigation (RCP 2.6) having a smaller aviation net NO_*x*_ RF than, lower mitigation scenarios (RCP 4.5, RCP 8.5) for the same aviation NO_*x*_ emission. Overall uncertainties are indicated by the grey shading, which is one standard deviation (68% confidence interval) from the ensemble of 20 NO_*x*_ studies presented in Supplementary Fig. 1.

The resulting RFs (Table 1) highlight that an aviation net NO_*x*_ RF can vary greatly depending on the background condition, and both anthropogenic surface and aircraft emissions affect the aviation net NO_*x*_ RF. At present, all scenarios predict increased aircraft NO_*x*_ emissions in the year 2050 that reach 2.17 Tg(N) year^-1^ for the low air-traffic growth and optimistic technology development and 5.59 Tg(N) year^-1^ for high air-traffic growth and low technology development: which compared with the year 2006, 0.71 Tg(N) year^-1^, are significant increases. In contrast, reductions of surface O_3_ precursor emissions are projected under each of the applied RCP scenarios for the year 2050, for which the cleanest background is predicted under RCP 2.6. The total aircraft net NO_*x*_ RF in 2006 is 3.5 mW m^-2^ and it increases to between 5.8 and 12.5 mW m^−2^ in 2050 for low- and high-growth scenarios, respectively; within each RCP scenario, the net aviation NO_*x*_ RF can differ by ~23%, depending on background conditions. The largest aviation net NO_*x*_ RF is observed under RCP 8.5 and the smallest for RCP 2.6 (for equal aviation NO_*x*_ emissions). This significant increase in the aviation net NO_*x*_ RF for 2050 is driven mainly by an intense rise of aviation NO_*x*_ emissions; however, reduced surface emissions resulting in a cleaner background atmosphere in the year 2050 to some extent mitigates the aviation impact (Fig. 1). For instance, if the anthropogenic surface emissions were kept constant at present levels, the 2050 aircraft net NO_*x*_ RF of the high air-traffic growth would be 17.5 mW m^−2^, that is 48% greater than under the 2050 RCP 2.6 and 28% greater than under 2050 RCP 8.5 background conditions.

**Table 1:** Aircraft NO_*x*_ radiative forcing (RF, mW m^−2^) for different background and aircraft emission scenarios in 2006 and 2050. Net NO_*x*_ RF is a sum of a short-term O_3_ (sO_3_), CH_4_, CH_4_-induced O_3_ (lO_3_) and SWV.

Emissions			RF, mW m^−2^				
	Background	Aircraft	sO_3_	CH_4_	lO_3_	SWV	Net NO_*x*_
2006 IPCC AR5	Base	REACT4C	14.8	−6.9	−3.4	−1.0	3.5
	−30% NO_*x*_		17.7	−9.5	−4.8	−1.4	2.0
	−30% CO		14.1	−6.7	−3.4	−1.0	3.0
	−30% NMVOC		13.9	−6.7	−3.4	−1.0	2.8
	−30% ALL		16.2	−8.5	−4.3	−1.3	2.2
2050	RCP 8.5	Low NO_*x*_ High Tech	45.9	−23.4	−11.7	−3.5	7.2
	RCP 4.5		43.7	−22.6	−11.3	−3.4	6.4
	RCP 2.6		40.6	−21.1	−10.5	−3.2	5.8
	RCP 8.5	High NO_*x*_ Low Tech	96.3	−50.8	−25.4	−7.6	12.5
	RCP 4.5		90.7	−48.6	−24.3	−7.3	10.6
	RCP 2.6		83.9	−45.3	−22.7	−6.8	9.2

This observed increase of aviation net NO_*x*_ RFs in the future is in agreement with other studies that have explored the climate impact from aviation NO_*x*_ emissions in 2050. Global CTMs and chemistry-climate models (CCMs) using 2050 aviation emissions derived from the Aviation Environmental Design Tool (AEDT) and the RCP 4.5 background scenario have been employed^18–20^. Unger et al.^19^ calculated that both the positive short-term O_3_ and negative CH_4_ RFs in 2050 increased by ~80% for the AEDT Base scenario (4.0 Tg(N) year^−1^), whereas Khodayari et al.^20^ estimated the 2050 short-term O_3_ RF to be 48–75% greater than in 2006, and the 2050 CH_4_ RF increased by 57–80%. From these studies, the available 2050 short-term O_3_ RF ranged from 30 to 162 mW m^−2^ and the CH_4_ RF varied from −36 to −72 mW m^−2^ (all estimates are for AEDT-Base and RCP 4.5 scenarios)^18,21^. The 90.7 and −48.6 mW m^−2^ calculated in this study with the RCP 4.5 background emissions are in line with estimates found in the literature. The existing spread in the calculated aviation NO_*x*_-induced effects is the result of both differences in the projections of aircraft emissions and the inter-model differences. The latter difference raises an important level of uncertainty^3^, for which the differences in model chemistry schemes and the treatment of physical processes play an important role. Also, due to the inclusion of more feedback processes and coupled interactions (particularly aerosol and cloud coupling processes), different responses between the offline models (CTMs) and the fully coupled models (CCMs) can be observed^18^. However, based on the reported net NO_*x*_ RFs available in the literature any systematic differences between CTMs and CCMs cannot be identified (Supplementary Fig. 1 and Supplementary Note 2).

### The impact of surface emissions on aviation net NO_*x*_ radiative forcing

**Fig. 2:**
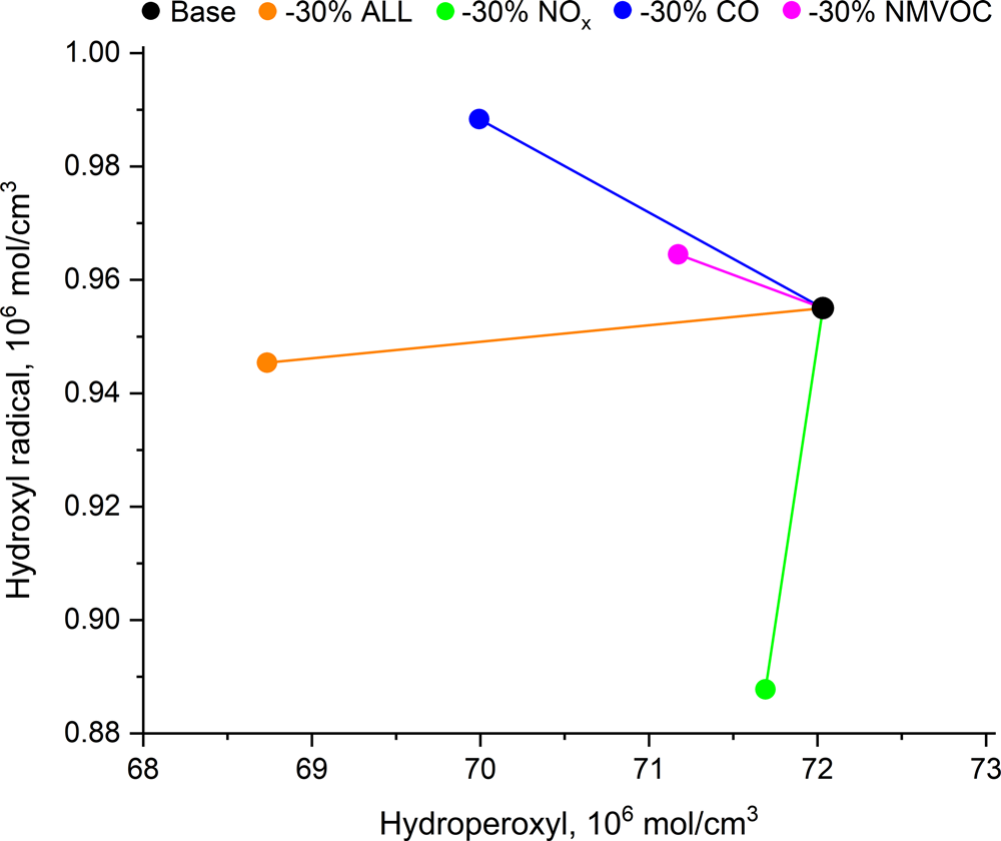
The oxidative capacity of the troposphere under different background conditions. By reducing emissions of individual precursors by 30% (NO_*x*_, CO, NMVOC), and all together (ALL) changes the concentrations of hydroxyl radical and hydroperoxyl. The oxidative capacity of the atmosphere to a great extent controls the abundance of most trace gas species, hence affecting CH_4_ lifetime and concentration of O_3_ (see text for details). Values are averaged globally within the vertical domain extending from surface to 100 hPa and modelled by MOZART-3 CTM.

Not only does the overall reduction of background emissions (i.e. different RCP scenarios) change the aircraft impact (same aircraft emissions), but also the mix of these reductions can have an impact. This is demonstrated by reducing individual precursors by −30% (NO_*x*_, CO, NMVOC), and all together (ALL), which changes the oxidative capacity of the atmosphere (Fig. 2). Figure 2 illustrates that a reduction of background NO_*x*_ emissions (alone) leads to a significant decrease in hydroxyl radical (OH) concentrations, while the reduction of CO and NMVOC emissions (individually) increases OH concentrations as their oxidation process becomes limited, leading to a reduced production of hydroperoxy radicals (HO_2_). As a result of the above, the reduction in surface NO_*x*_ emissions increases CH_4_ lifetime and decreases the concentration of tropospheric O_3_, while the reductions of CO and NMVOC cause the decrease of both tropospheric O_3_ and CH_4_, an observation that is consistent with other studies^5,22^. These dependencies are observed not only near the ground but also in the upper troposphere–lower stratosphere (UTLS) region, where most of aviation emissions occur; therefore, affecting aviation effects (Supplementary Fig. 2). In general, reducing surface emissions of NO_*x*_, CO and NMVOC individually decreases the net aviation NO_*x*_ RF by up to 43%, the most effective being a reduction in surface NO_*x*_ emissions alone, and the least effective being a reduction in CO, which results in a reduction of aviation net NO_*x*_ RF of 14% (Table 1). A simultaneous reduction of all surface emissions by 30% decreases the aviation net NO_*x*_ RF by 37% (for the same aircraft emissions). The reduction of surface NO_*x*_ has the greatest potential in affecting the aviation net NO_*x*_ RF; any 1% change of surface NO_*x*_ emissions, modifies aircraft net NO_*x*_ RF by ~1.5% (this estimate is inventory-dependant and here it varies from 1.4% to 1.6% for REACT4C 2006 data and high NO_*x*_ 2050 scenario, respectively).

**Fig 3:**
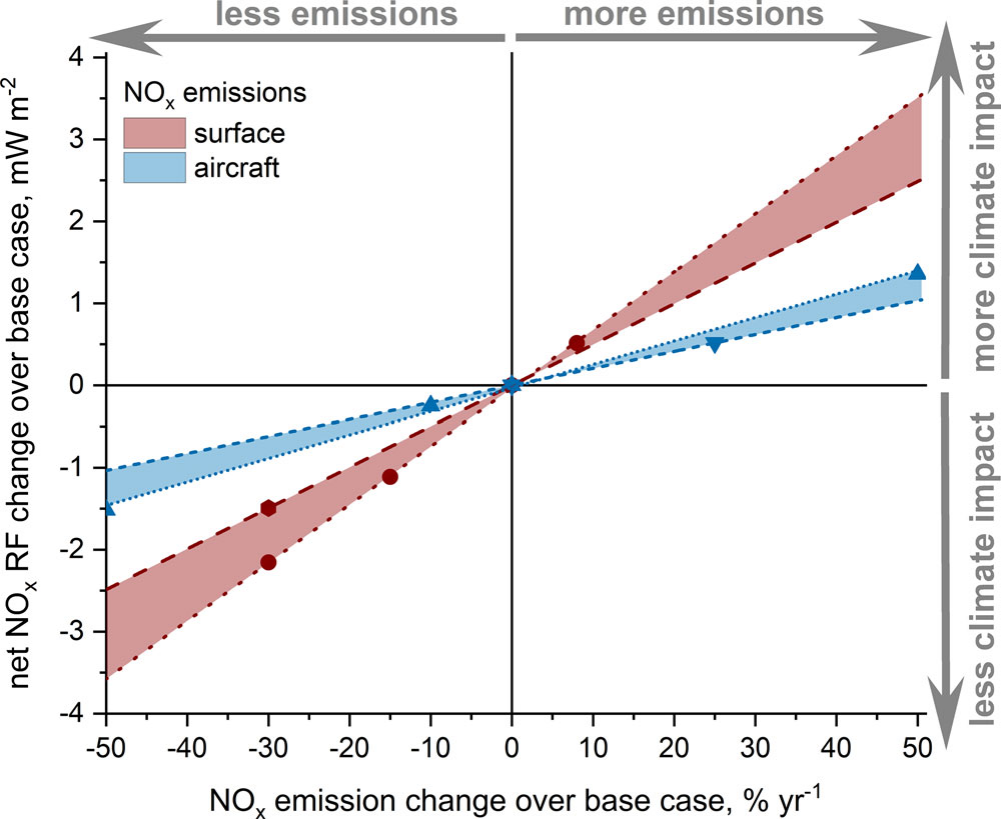
Aviation net NO_*x*_ radiative forcing change versus percentage change in NO_*x*_ emission rates from surface and aviation sources. The aviation NO_*x*_ response has been explored for varying, both, surface (red) and aviation (blue) emissions (dots are individual experiments, lines are the best fit lines). In the red case whilst surface NO_*x*_ emissions are changing, aircraft NO_*x*_ emissions are kept constant and this has been analysed for the highest and lowest projected range of aircraft NO_*x*_ emissions, 2050 HighNO_*x*_-LowTech scenario (red dashed line; *n* = 2, *r*^2^ = 1.00) and 2006 REACT4C (red dotted line; *n* = 4, *r*^2^ = 1.00, *p* < 0.05). In the blue case whilst aircraft NO_*x*_ emissions are changing, the surface NO_*x*_ emissions are kept constant and this has been analysed for the highest and lowest projected range of surface NO_*x*_ emissions, 2005 IPCC AR5 (blue dotted line; *n* = 4, *r*^2^ = 0.998, *p* < 0.05) and 2050 RCP 2.6 (blue dashed line; *n* = 3, *r*^2^ = 0.998, *p* < 0.05). The exact experiments that are used here are presented in Supplementary Table 1.

There is a well-known increase in O_3_ production (per unit of emitted N) as background NO_*x*_ levels decrease and this is what we observe here as well. However, we also calculate a strong dependence of aircraft CH_4_ lifetime reduction on surface emissions that becomes more efficient with decreasing NO_*x*_. So, for a cleaner NO_*x*_ background the positive short-term O_3_ RF increases, as expected, but the associated CH_4_ RF (and all the CH_4_-induced RFs) reduction increases even more, explaining why the net NO_*x*_ RF decreases, rather than increasing^3^ for a cleaner NO_*x*_ background. This strong CH_4_ response is possibly triggered by increased CH_4_ lifetime due to reduced oxidative capacity (Fig. 2). The 30% reduction of surface NO_*x*_ increases the positive short-term O_3_ RF by 16%; however, the magnitude of the negative long-term CH_4_ RF increases even more, by 28%. Thus, less background NO_*x*_ reduces the net NO_*x*_ effect from aviation (for the same aviation NO_*x*_ emissions). Moreover, it turns out that decreasing surface NO_*x*_ emissions plays a larger role in reducing the aviation net NO_*x*_ RF than decreasing aircraft NO_*x*_ emissions (Fig. 3) in percentage terms. Figure 3 shows a steeper slope in the reduction of net NO_*x*_ RF from percentage changes in surface emissions than from aviation emissions themselves. For example, in order to reduce the global climate impact of aviation NO_*x*_ by 1 mW m^−2^, a 17% reduction in present levels of surface NO_*x*_ emissions is needed; in the case of aviation NO_*x*_, it requires the reduction of emissions by 35%. Reducing aviation NO_*x*_ emissions by such a large amount (35%) for, e.g. a 1 W m^-2^ net NO_*x*_ RF reduction could be quite technologically challenging and have a strong risk of increasing aircraft CO_2_ emissions with a potentially perverse total RF outcome^10^. If a scenario is envisaged of falling surface NO_*x*_ emissions, reducing aircraft NO_*x*_ emissions at the expense of either missed opportunities to reduce CO_2_ emissions or even actually increasing CO_2_ emissions could be exactly the wrong thing to do and induce perverse climate outcomes.

## Discussion

**Fig. 4:**
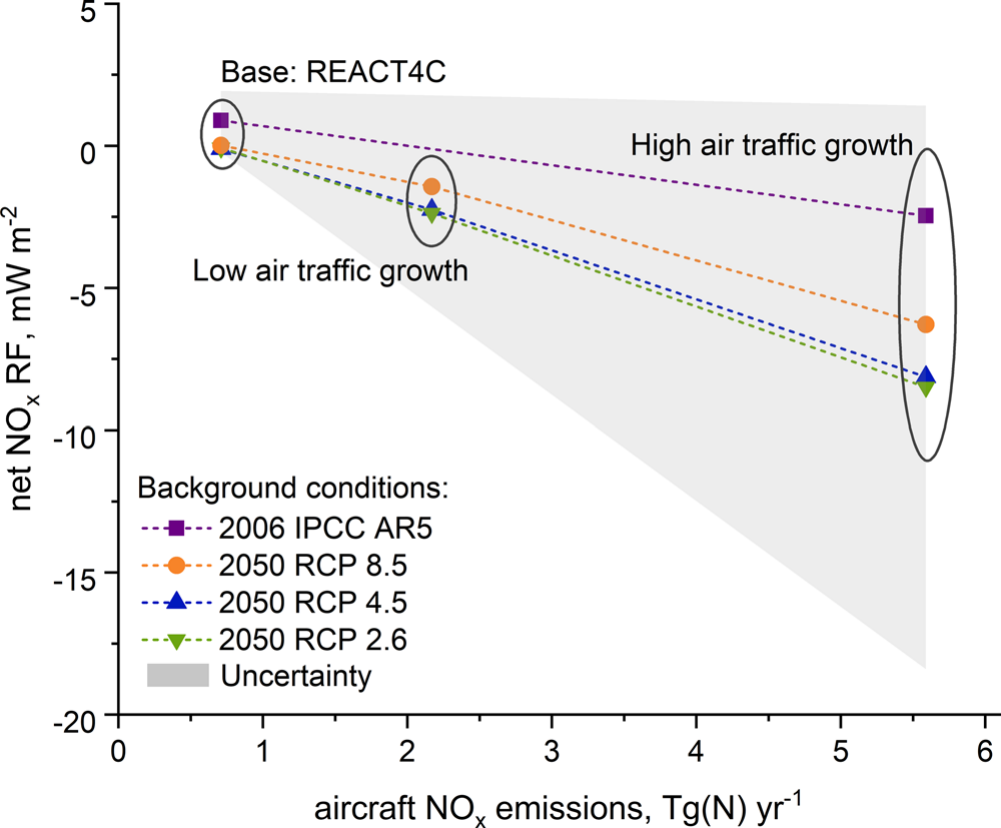
Net NO_*x*_ radiative forcing by emission rate, updated CH_4_ parameterisation. Aviation net NO_*x*_ radiative forcing (RF) (the sum of the short-term positive O_3_ RF perturbation and the negative RF terms caused by a reduction in CH_4_ lifetime, see Methods), by aviation NO_*x*_ emission rate according to a range of background emission scenarios, utilising the updated simplified expression for the calculation of CH_4_ forcing of Etminan et al.^23^ Aviation net NO_*x*_ RF systematically decreases with increasing NO_*x*_ emissions from aviation, showing a variation according to the background surface emissions, with high mitigation (RCP 2.6) having a smaller aviation net NO_*x*_ RF than, lower mitigation scenarios (RCP 4.5, RCP 8.5) for the same aviation NO_*x*_ emission. The pattern of behaviour is in contrast to Fig. 1 because the updated CH_4_ forcing expression accounts for the short-wave forcing of CH_4_, increasing CH_4_ RF estimates by approximately 25%, which in the case of aviation net NO_*x*_ impacts greatly increases the negative terms from the reduction in CH_4_ lifetime from aviation NO_*x*_, tipping the net NO_*x*_ term from being positive to negative with increasing aviation NO_*x*_ emissions. Overall uncertainties are indicated by the grey shading, which is one standard deviation (68% confidence interval) from the ensemble of 20 NO_*x*_ studies presented in Supplementary Fig. 1.

The short-term O_3_ RF is very sensitive to changes in all explored changes in surface precursor emissions (NO_*x*_, CO and NMVOC). The long-term CH_4_ RF is mainly affected by the reduction in surface NO_*x*_ emissions and it changes very little in the case of the reductions of surface CO and NMVOC alone (probably due to the decrease in OH consumption by CO). This is in agreement with responses from other sensitivity tests performed with UCI CTM by Holmes et al.,^3^ with the difference that our short-term aviation O_3_ RF is not as responsive to surface CO emissions as those modelled with the UCI CTM. In addition, after accounting for the long-term negative RFs that were not given in their 2011 paper (long-term O_3_ and reductions in stratospheric water vapour, SWV), they also observe that the reduction in surface NO_*x*_ emissions decreases the RF from aviation NO_*x*_, a 42% reduction in the aviation net NO_*x*_ RF resulting from a halving of surface NO_*x*_ emissions (C. D. Holmes, personal communication, October 19, 2018), which is in reasonable agreement with the sensitivity shown here. In general, to a great extent, the long-term CH_4_-mediated effects drive the response of aviation net NO_*x*_ RF resulting from modified NO_*x*_ emissions. Taking into account that these long-term RFs are fully parametrised as well as the fact that the CH_4_/O_3_ ratio is very model specific^4^ make the impact of surface NO_*x*_ emissions on aircraft net NO_*x*_ RF relatively more uncertain than the impact of other O_3_ precursor emissions. For example, if the new CH_4_ RF simplified expression that accounts for short-wave forcing is used^23^, reduction in surface NO_*x*_ emissions not only decreases the aviation net NO_*x*_ RF but also changes its sign from positive to negative. Table 2 gives the recalculated aviation RF numbers from Table 1 using a simplified expression for RF of CH_4_ as presented by Etminan et al.^23^ The improved understanding of CH_4_ RF has a significant impact on aviation estimates as it increases the negative CH_4_ RF from aviation NO_*x*_ emissions by ~20%, which substantially reduces the aircraft net NO_*x*_ RFs (Table 2). Moreover, the revision to the CH_4_ term provides a perspective that as aviation NO_*x*_ emissions are reduced an *increase in* the global aviation net NO_*x*_ RF is shown, and vice versa (Fig. 4 and Table 2). This revised formulation of the CH_4_ for RF does not contradict findings presented in this study, in terms of the sensitivity of responses, but turns out to be crucial for quantification of net NO_*x*_ RFs and it provides a new perspective on the potential RF impact from future aviation NO_*x*_ emissions. Other potential effects from NO_*x*_ emissions include the direct enhancement of nitrate aerosol and indirect formation of sulfate aerosol (more efficient conversion of sulfur dioxide to sulfuric acid via increased OH). These aerosol effects are associated with large uncertainties and are addressed in only a few modelling studies^24,25^ and were not considered here. However, the effects of NO_*x*_ on aerosol abundances are expected to result in negative forcings, such that inclusion of these processes would increase the negative NO_*x*_-associated forcings and be consistent with the findings of this work that emphasizes the role of the negative forcings.

**Table 2:** The recalculated aircraft NO_*x*_ radiative forcing (RF) from Table 1 using a revised simplified expression for the RF of CH_4_ as presented by Etminan et al.^23^.

Emissions			RF, mW m^−2^				
	Background	Aircraft	sO_3_	CH_4_	lO_3_	SWV	Net NO_*x*_
2006 IPCC AR5	Base	REACT4C	14.8	−8.4	−4.2	−1.3	0.9
	−30% NO_*x*_		17.7	−11.7	−5.9	−1.8	−1.6
	−30% CO		14.1	−8.2	−4.1	−1.2	0.5
	−30%NMVOC		13.9	−8.3	−4.1	−1.2	0.3
	−30% ALL		16.2	−10.5	−5.2	−1.6	−1.1
2050	RCP 8.5	Low NO_*x*_ High Tech	45.9	−28.7	−14.3	−4.3	−1.4
	RCP 4.5		43.7	−27.8	−13.9	−4.2	−2.3
	RCP 2.6		40.6	−26.0	−13.0	−3.9	−2.4
	RCP 8.5	High NO_*x*_ Low Tech	96.3	−62.2	−31.1	−9.3	−6.3
	RCP 4.5		90.7	−59.9	−29.9	−9.0	−8.1
	RCP 2.6		83.9	−56.0	−28.0	−8.4	−8.5

These new results (Table 2 and Fig. 4) show that as a generality, the net NO_*x*_ RF from aviation decreases with air-traffic growth and corresponding increased aviation NO_*x*_ emissions with reduced background emissions. The predicted cleaner background in the future acts with these reduced net NO_*x*_ RFs. Therefore, it is worth highlighting that the ongoing efforts in cutting ground-level air pollution serve not only air quality improvements but are also beneficial for reducing the climate impact of aviation NO_*x*_ emissions.

Climate change is considered to affect tropospheric chemistry. The modified oxidising capacity is expected to influence methane lifetime such that in a wetter and warmer climate it might either shorten or increase^26,27^ depending on the scenario. Also, on one hand, the increased water vapour might lead to increased O_3_ destruction in the tropics, whereas on the other, enhanced stratosphere–troposphere exchange could increase the net O_3_ flux to the troposphere^14,28^. The impact of the physical aspects of climate change on tropospheric composition is complex and is still highly uncertain. The inclusion of an interactive climate in the experiments might affect the results presented in this study. However, it is not expected that the current findings would become irrelevant, since it is the emission scenarios that to a great extent affect the future evolution of tropospheric chemistry^29,30^. The decreases in surface O_3_ precursor emissions over present-day values presented in RCP dataset are consistent with most other future emission scenarios that consider even more extensive air quality legislation, e.g. ECLIPSE (Evaluating the CLimate and Air Quality ImPacts of Short-livEd Pollutants) or SSPs (Shared Socioeconomic Pathways) databases. This is in stark contrast to aviation emissions, for which strong growth is predicted and the global civil fleet may more than double within the next 20 years, from ~21,000 aircraft in 2018 to ~48,000 aircraft in 2037^31^. Whilst there is a possibility for the net NO_*x*_ RF to be weakened due to a cleaner background, this is not as simple for an aviation CO_2_ RF. CO_2_ accumulates in the atmosphere due to its fractional millennial timescale which means that its climate effect is determined by the cumulative emissions over time. As a consequence, the RF in 2018 from aircraft CO_2_ emissions is around twice greater than that from aircraft NO_*x*_ emissions^32^. However, what is even more important is the difference in the nature of their responses (Supplementary Fig. 3). At present, the temperature response from a unit emission of aircraft NO_*x*_ is the strongest in the year of emission and it diminishes thereafter. Moreover, after around 15 years it changes the sign from positive (warming) to negative (cooling) to disappear after around 60 years. On the contrary, the emission of CO_2_ leads to a uniform positive (warming) temperature response from increased atmospheric levels of this gas and after a 100 years, the unit emission emitted in the year 1 still provides a significant positive signal (as modelled by the simple climate model, LinClim, see Methods).

There are various measures to reduce fuel demand (and therefore CO_2_ emissions) such as market-based measures or stricter aircraft CO_2_ emission standards; the latter, as it is associated with trade-off between aviation CO_2_ and NO_*x*_ might raise some dilemmas. In view of the low impact of reducing aviation NO_*x*_ any potential trade-offs with CO_2_ should not be risked and also any potential savings in CO_2_ should not be forsaken in the pursuit of lower NO_*x*_ in terms of climate protection^10^. We acknowledge the necessity to reduce aircraft NO_*x*_ emissions for local air quality benefits; the source apportionment in any given location is likely to be unique, depending on volume of air traffic and other local sources. However, the aircraft-related emissions of NO_*x*_ are of clear importance for many locations. From a climate benefit point of view, we suggest that any vision of more stringent NO_*x*_ regulations needs to be revisited, as it might be more worthwhile to concentrate more on CO_2_ reductions at the cost of NO_*x*_, not vice versa, especially in the light of necessary forthcoming decarbonisation to avoid an^33^ increase of 1.5°. Coherent comparative assessments that would consider both climate and air quality impacts are needed. There are just a few studies that try to tackle this issue^34–36^ and none that would consider these aspects under changing background conditions.

The CO_2_ emissions still provide the majority of the long-term warming (if not the instantaneous RF) from aviation, and a smaller change in its emission affects the total forcing much more than an equivalent change in NO_*x*_ emission. The mitigation of non-CO_2_ effects is scientifically uncertain and trading against CO_2_ could produce perverse outcomes^10^, the climate benefits from any reduction of aviation CO_2_ emissions are indisputable.

## Methods

### Chemistry transport model and emission data

The model for ozone and related chemical tracers, version 3 (MOZART-3) was used for this study. This is a 3D CTM that has been evaluated by Kinnison et al.^16^ and used for an extensive range of different applications^37,38^, including studies dealing with the impact of aircraft NO_*x*_ emissions on atmospheric composition^39,40^.

MOZART-3 accounts for advection based on the flux-form semi-Lagrangian scheme^41^, shallow and mid-level convection^42^, deep convective routine^43^, boundary layer exchanges^44^, or wet and dry deposition^45,46^. MOZART-3 reproduces detailed chemical and physical processes from the troposphere through the stratosphere, including gas-phase, photolytic and heterogeneous reactions. The kinetic and photochemical data are based on the NASA/JPL evaluation^47^.

The model configuration used in this study includes a horizontal resolution of T42 (~ 2.8° × 2.8°) and 60 hybrid layers, from the surface to 0.1 hPa. The transport of chemical compounds is driven by the meteorological fields from the European Centre for Medium Range Weather Forecast (ECMWF), 6-h reanalysis ERA-Interim data for the year 2006^48^. This meteorological conditions were used for all runs, including the 2050 simulations.

The aviation NO_*x*_ emissions for the years 2006 and 2050 were determined based on the REACT4C base case dataset (CAEP/8 movements)^39^ and ICAO-CAEP^49^ aviation emission projections, respectively. Emissions of aircraft NO_*x*_ were calculated to be 0.71 Tg(N) year^-1^ in 2006 and 2.17 Tg(N) year^-1^ in the 2050 low air-traffic growth and optimistic technology-development scenario and 5.59 Tg(N) year^-1^ in the 2050 high air-traffic growth and low technology-development scenario. The 2050 aviation scenarios were chosen to represent the highest and lowest projected range of possible aircraft NO_*x*_ emissions in 2050 from data derived from the ICAO-CAEP trends work^49^. First, three aviation traffic demand forecasts out to 2040 are produced (a low, central and high traffic-demand scenario) these demand scenarios are translated by ICAO-CAEP to fleet scenarios, fuel efficiency scenarios are then superimposed upon the fleet scenarios to produce a range of technology and operational improvement scenarios ranging from a technology freeze, through to low, moderate, advanced and optimistic improvement scenarios. Extrapolation of the fuel burn 2040 results out to 2050 is also undertaken and reported by the ICAO-CAEP. Further to the fuel efficiency and traffic demand assumptions, two separate NO_*x*_ scenarios were developed by ICAO-CAEP resulting in the derivation of two future 2050 fleetwide NO_*x*_ emission indices (EINO_*x*_ in terms of grams of NO_*x*_ per kilogram of fuel burned): a high and a low EINO_*x*_. In this study the high and low EINO_*x*_ values for the future fleet are applied to the range of estimates of fuel burn in 2050 to calculate a corresponding range of NO_*x*_ emission estimates in 2050. The range of NO_*x*_ estimates in 2050 varies from the low NO_*x*_ scenario of 2.17 Tg(N) year^-1^ (low EINO_*x*_, the low traffic demand with the more efficient optimistic fuel burn scenario, i.e. low fuel burn estimate) and the high NO_*x*_ estimate of 5.59 Tg(N) year^-1^ (high EINO_*x*_, the high traffic demand forecast and the low fuel efficiency scenario, i.e. higher fuel estimate).

The present-day surface (non-aviation) emissions (base) represented year 2005. The anthropogenic and biomass burning emissions were taken from IPCC AR5^50^ and the biogenic emissions were taken from POET^51^. Four different cases were investigated: global reduction of surface NO_*x*_ emissions (−30% NO_*x*_), global reduction of surface CO emissions (−30% CO), global reduction of NMVOC emissions (−30% NMVOC) and global reduction of ALL these species simultaneously (−30% NO_*x*_, CO, NMVOC). All other sources of emissions, including aircraft NO_*x*_ emissions were held constant for each experimental case. The 2050 gridded surface emissions (anthropogenic and biomass burning) were determined by Integrated Assessment Models (IAMs) for the three Representative Concentration Pathways^52^ (RCPs): a high mitigation scenario that forecast the smallest impact to climate (RCP 2.6), business-as-usual scenario (RCP 4.5) and high climate impact scenario (RCP 8.5). Concentrations of long-lived chemical species and greenhouse gases were based on the RCP emissions, converted to concentrations by Meinshausen et al.^53^ Natural emissions (e.g. isoprene, lightning and soil NO_*x*_ or oceanic emissions of CO) were not specified in these future scenarios; thus, they were not modified here and were kept the same for all the simulations. The parametrisation of NO_*x*_ emissions from lightning was defined as a function of the location of convective cloud top heights^54,55^ and their global source were calculated to be 4.7 Tg(N) year^−1^.

The series of sensitivity experiments were performed in order to have a broad perspective on how aircraft and background emissions might affect net NO_*x*_ climate impact. The detailed list of simulations exploited in this study shows Supplementary Table 1.

### Radiative forcing calculations

The Edwards-Slingo radiative transfer model^56^ (RTM) was used for the calculation of the forcing associated with aviation NO_*x*_-induced short-term O_3_. The monthly O_3_ fields from MOZART-3 were converted into mass mixing ratios and interpolated onto RTM vertical and horizontal resolution. The applied RTM is an offline version of the UK Met Office Unified Model and it calculates the radiative fluxes and heating rates based on the δ-Eddington of the two-stream equations in both, the long-wave (9 bands) and short-wave (6 bands) spectral regions. Cloud treatment is based on averaged International Satellite Cloud Climatology Project (ISCCP) D2 data.^57^ Climatological fields of temperature and specific humidity are based on ERA-Interim data^48^. In terms of the 2050 RF calculations, the concentrations of long-lived species were modified according to specific RCP scenarios^53^ and were consistent with MOZART-3 set up.

The CH_4_ concentrations change is assumed to be in equilibrium with the OH change due to the aircraft NO_*x*_ perturbation from constant emissions^58^. Since CH_4_ mixing ratios were prescribed as a lower boundary condition and the simulations were not long enough, the steady-state CH_4_ concentration ([CH_4_]_ss_) was calculated from the change in its lifetime with respect to reaction with tropospheric OH derived from MOZART-3 simulations as shown in Eq. 1:

1 $$\left[ {{\mathrm{CH}}_4} \right]_{{\mathrm{ss}}} = \left[ {{\mathrm{CH}}_4} \right]_{{\mathrm{ref}}} \times (1 + 1.4 \times {\Delta} \tau _0/\tau _{{\mathrm{ref}}})$$

A factor of 1.4 was used to reflect the CH_4_ feedback on its own lifetime^3,58^. Here *τ*_ref_ denotes the lifetime of CH_4_ versus reaction with OH in the unperturbed simulation, Δ*τ*_0_ the change in CH_4_ lifetime between the unperturbed simulation and the NO_*x*_-perturbed simulation and [CH_4_]_ref_ the CH_4_ mixing ratio in the unperturbed simulation.

This steady-state CH_4_ aircraft response was further used for the CH_4_ RF calculations using, as in IPCC AR5^59^, a simplified expression (Eqs. 2 and 3) originally defined in Myhre et al.^60^

2 $${\Delta} F = 0.036(\sqrt M -\sqrt {M_0} )-\left( {f\left({M,N_0} \right)-f\left( {M_0,N_0} \right)} \right)$$

3 $$f\left( {M,N} \right) = 0.47{\mathrm{ln}}[1 + 2.01 \times 10^{ - 5}\left( {MN} \right)^{0.75} +\, 5.31 \times 10^{ - 15}M\left({MN} \right)^{1.52}]$$

where *N* is N_2_O in ppbv, *M* is CH_4_ in ppbv and subscript 0 denotes unperturbed concentrations.

Long-term O_3_ caused by CH_4_ changes was calculated according to IPCC AR5^59^, as 50% of the CH_4_ RF. Modified CH_4_ also affects SWV and give an additional RF of 15% of CH_4_ RF^61^.

The recalculated aviation RF estimates presented in Table 2 are based on the same methodology as original values shown in Table 1 and as described above. The only differences comes from the application of the new simplified expression for RF of CH_4_^23^ as shown in Eq. 4 that replaces the old expression presented in Eqs. 2 and 3:

4 $${\Delta} F = {[a {\times} {\bar {M}} + b {\times} {\bar {N}} + 0.043]} {\times} ({\sqrt {M}} - {\sqrt {M_0}} )$$

where *a* = −1.3 × 10^−6^ Wm^−2^ ppb^−1^, *b* = −8.2 × 10^−6^ Wm^−2^ ppb^−1^, *M* and *N* are concentrations of CH_4_ and N_2_O, respectively, and subscript 0 denotes unperturbed concentrations. For terms within the square brackets, the gas concentrations are the mean of the unperturbed and perturbed concentrations, e.g. $\bar M$ = 0.5 × (*M* + *M*_0_).

### The temperature responses from a unit aircraft CO_2_ vs NO_*x*_ emissions

The time scales of the climate effects of CO_2_ and NO_*x*_ are very different and these processes of the long-term accumulation vs short-term disappearing, respectively, were explored here. The responses presented on Supplementary Fig. 2 are based on a pulse emission of a 1 Tg(N) year^-1^ in year 1.

In order to observe the temperature response from a unit emissions of aircraft NO_*x*_ the methodology presented by Aamas et al.^62^ have been applied and Absolute Global Temperature change Potentials (AGTP) have been calculated based on steady-state CTM/RTM results (base case). The forcing for NO_*x*_ is assumed to be a result of a pulse that lasts for 1 year followed by an exponential decay of the resulting forcing from the end of the year 1 onwards. The NO_*x*_ effect is the sum of the short-term O_3_ effect, CH_4_ (with SWV) effect and CH_4_-induced O_3_ effect, and there is a perturbation from the forcing for *t*  < 1 (this determines the temperature response of the emissions that occur in the first year) and from the forcing for *t* ≥ 1 (this determines the temperature response of atmospheric perturbation lasting past one year). Thus, the total AGTP_NO__*x*_ (provided that the time horizon (*H*) is >1) is the sum of ${\mathrm{AGTP}}_{{\mathrm{NO}}x}^{t < 1}$(H) and ${\mathrm{AGTP}}_{{\mathrm{NO}}x}^{t \ge 1}$(H):

- For perturbation from RF occurring *t* < 1
  5 $${\mathrm{AGTP}}_{{\mathrm{NO}}x}^{t < 1}( H ) = \; {\Delta} F_{{\mathrm{NOx}}}^{{\mathrm{SS}}}\mathop {\sum }\limits_{j = 1}^2 \left\{ c_j\left[ \exp \left( {\frac{{1 - H}}{{d_j}}} \right) - \exp {\left( { - \frac{H}{{d_j}}} \right)} \right] \right. \\ \left.+\; \frac{{c_j\tau }}{{\tau - d_j}}\left[ \exp \left( { - \frac{H}{{d_j}}} \right) - \exp \left( {\frac{{1 - H}}{{d_j}}} \right)\exp {\left( { - \frac{1}{\tau }} \right)} \right] \right\}$$
- For perturbation from RF occurring *t* ≥ 1

6 $${\mathrm{AGTP}}_{{\mathrm{NO}}x}^{t \ge 1}\left( H \right) = {\Delta} F_{{\mathrm{NO}}x}^{{\mathrm{SS}}}\left[ {1 - \exp \left. {\left( { - \frac{1}{\tau }} \right)} \right]} \right.\mathop {\sum }\limits_{j = 1}^2 \frac{{c_j\tau }}{{\tau - d_j}}\left[ {\exp \left( {\frac{{1 - H}}{\tau }} \right) - \exp \left. {\left( {\frac{{1 - H}}{{d_j}}} \right)} \right]} \right.$$

The superscript SS indicates steady-state, *τ* is the lifetime and it is the short-lived lifetime (*τ*_s_) for short-term O_3_ (here it is 0.267) whilst the primary-mode lifetime (*τ*_pm_) characterises CH_4_ and CH_4_-induced O_3_ (for the MOZART-3’s base case it is 12.02 year). The c_j_ are the components of climate sensitivity and d_j_ are the corresponding time scales and these values are taken from Boucher and Reddy^63^.

In order to observe the temperature response from a unit emission of aircraft CO_2_ a simple climate model (SCM), LinClim was utilised. LinClim is a linear climate response model that has been customised specifically to aviation^10,64,65^. It uses a single impulse response function that is calibrated against more sophisticated parent model. Aviation fuel data are used to calculate CO_2_ emissions that is then applied in the linear response function from Hasselmann et al.^66^ in order to derive CO_2_ concentrations. The carbon cycle in LinClim is based on the Maier-Reimer and Hasselmann^67^ model and the CO_2_ RF is calculated using the function applied in IPCC AR4^68^. The temperature response formulation is based on the method presented by Hasselmann et al.^69^ The calculated temperature response is also dependent on the climate sensitivity parameter and the lifetime of the temperature perturbation that are tuned to LinClim’s parent General Circulation Model (GCM), here it is ECHAM4.

Here the LinClim was used to calculate the temperature response from a pulse/unit emission of aviation CO_2_ over the long time period. The background scenario chosen was represented by RCP 8.5^53^ as its global emissions are the closest to the current levels of CO_2_ in the atmosphere. The amount of aircraft NO_*x*_ that is produced from the fuel burned is described by the emission index (EINO_*x*_). The current EINO_*x*_ is 15.14 g(NO_2_)/kg(fuel)^49^ and it has been applied here. Knowing that for every 1 kg of fuel used, 3.16 kg of CO_2_ is emitted, the 1 Tg of emitted N is equivalent to 217 Tg fuel and therefore, 686 Tg of emitted CO_2_. This emission was released as a pulse in the year 1 and the consequent CO_2_ temperature response was observed for the following 100 years.

### Reporting summary

Further information on research design is available in the Nature Research Reporting Summary linked to this article.

## Supplementary information

Supplementary Information

Peer Review File

Reporting Summary

## Data Availability

All data discussed in the manuscript and Supplementary Information are presented in Source Data. All data generated for this study (2006 and 2050 CTM and RTM simulations) are available on request from the corresponding author. Source data are provided with this paper.

## Source data

Source Data

## Code availability

The modelling data have been post-processed using IDL 8.5.1, and all the scripts are available on request from the corresponding author.
