# Supplementary material for: Greater fuel efficiency is potentially preferable to reducing NOx emissions for aviation’s climate impacts
Source: Nat Commun. 2021 Jan 25;12:564. doi: 10.1038/s41467-020-20771-3 (PMC7835228; doi:10.1038/s41467-020-20771-3)
Supplement: Supplementary file 1 — Supplementary Information [file 41467_2020_20771_MOESM1_ESM.pdf]

Greater fuel efficiency is potentially preferable to reducing NO_x emissions for aviation's climate impacts

Agnieszka Skowron, David S. Lee, Rubén Rodríguez De León, Ling L. Lim, Bethan Owen

Supplementary Tables

Supplementary Table 1: The list of performed simulations. The bolded entries present experiments that have been exploited in Figure 3.

		Aircraft emissions / Surface emissions	Aircraft NO_x emissions (Tg(N) yr^{-1})						
			2006					2050	
			REACT4C (0.71)	-50% REACT4C (0.35)	-10% REACT4C (0.64)	+25% REACT4C (0.89)	+50% REACT4C (1.06)	low air growth (2.17)	high air growth (5.59)
Background conditions	2006	IPCC AR5	**X**	**X**	**X**	**X**	**X**		**X**
		+10% NO_x	**X**						
		-15% NO_x	**X**						
		-30% NO_x	**X**						**X**
		-30% CO	X						
		-30 NMVOC	X						
		-30% ALL	X						
	2050	2050 RCP 8.5	X			X		X	X
		2050 RCP 4.5	X					X	X
		2050 RCP 2.6	**X**			**X**		**X**	X

Supplementary Figures

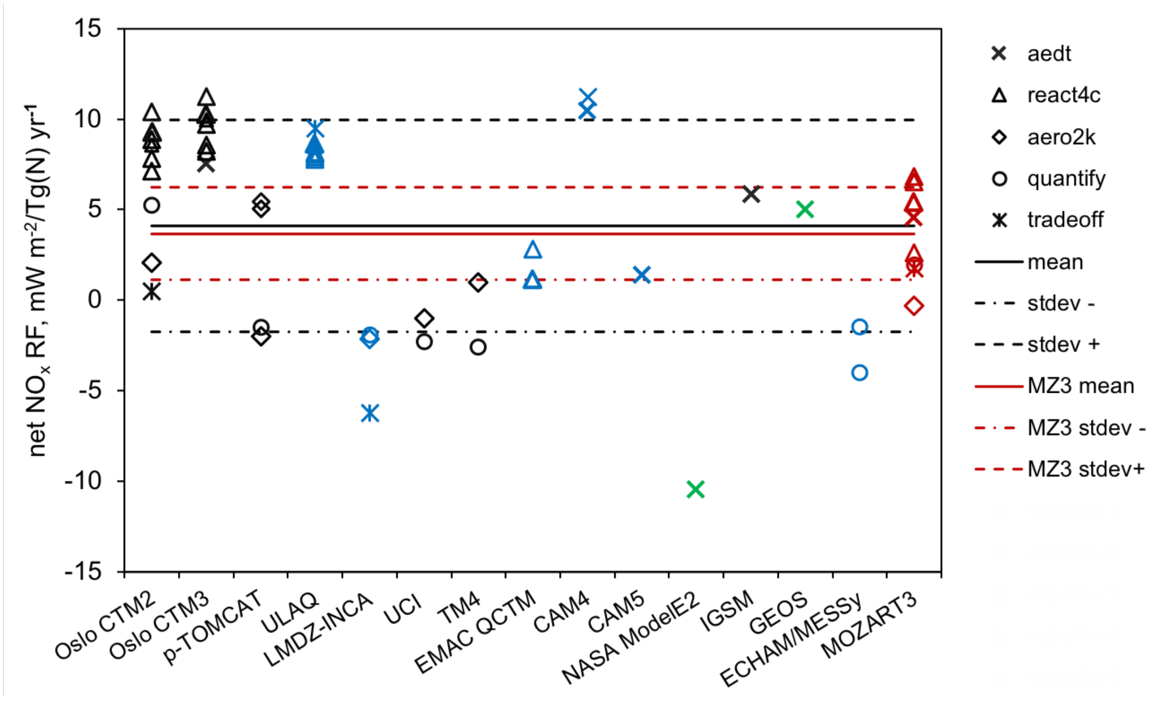

Supplementary Figure 1: The results of the global annual net NO_x RFs from aircraft studies that have been published since the IPCC (1999) Special Report 'Aviation and the Global Atmosphere'. The analysis covers a wide range of global atmospheric chemistry/climate models (black – CTMs, blue – CCMs in CTM mode, green – CCMs) and present-day aviation emission inventories (different shapes of data points, see key).
The CTM MOZART-3 numbers have been marked in red to show where it stands among other models. Each point represents a particular model study, solid lines denote the mean value, dashed lines show the one standard deviation range of results. The detailed list of studies is given in Lee et al.[1]

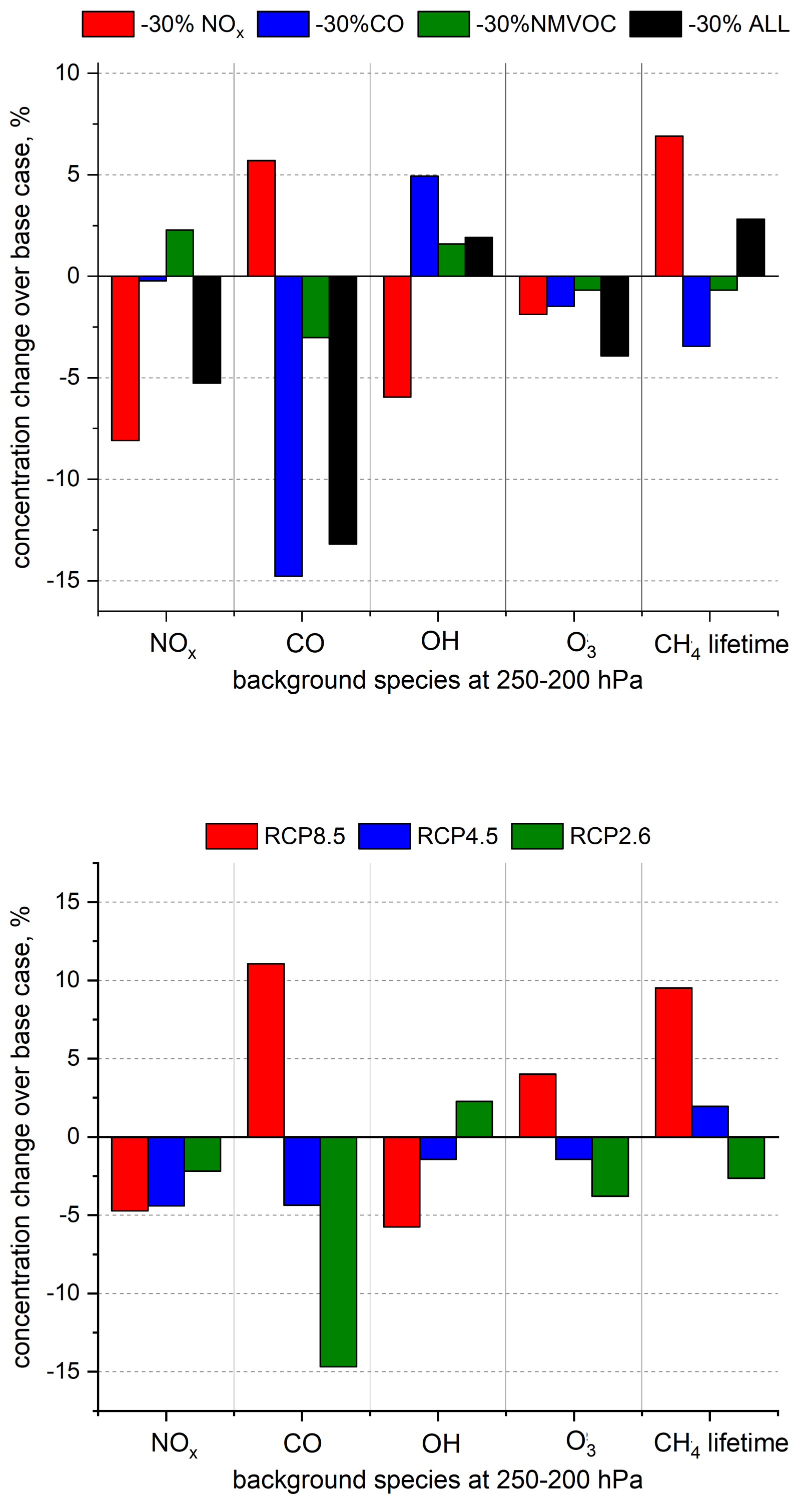

Supplementary Figure 2: Percentage changes in concentrations of NO_x, CO, OH, O_3 at aircraft cruise altitudes between 250 and 200 hPa, and the percentage change in CH_4 lifetime, between a base case and changes in surface emissions for different species (upper panel) and scenarios (lower panel) as modelled by MOZART-3.

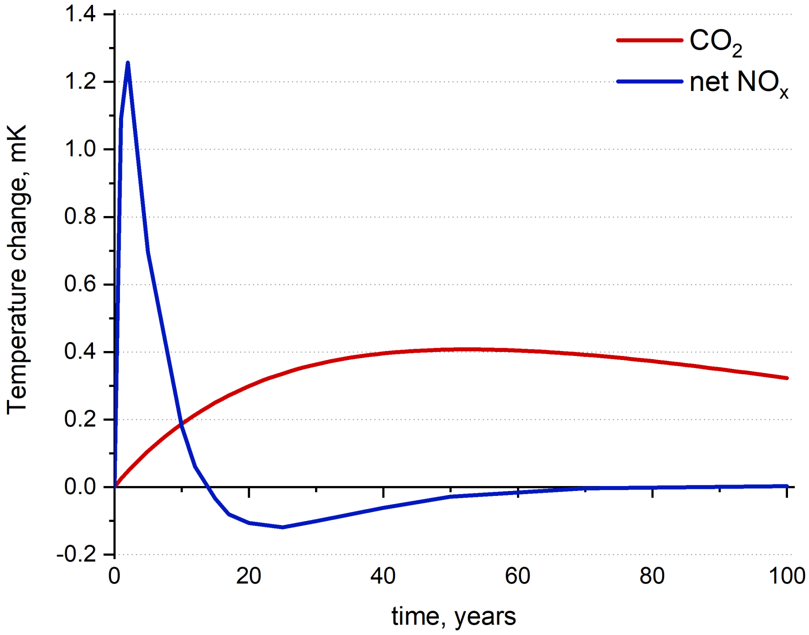

Supplementary Figure 3: The temperature response from a fleet-sized pulse of aviation CO_2 (red line) and corresponding NO_x (blue line) emissions in year 1 over 100 years (see Methods for details).

Supplementary Note 1: Comparison of modelled, by 3D CTM MOZART-3, atmospheric constituents with measurement data

The ability of MOZART-3 to represent atmospheric processes and constituents was extensively evaluated by Kinnison et al.[2] and was shown in a number of publications[3,4], with special attention paid to the upper troposphere and lower stratosphere region (UTLS)[5-8]. Through these publications, the capability of MOZART-3 in reproducing atmospheric composition, both globally and seasonally, with relatively good accuracy was shown. However, while the chemical tropopause exchanges are qualitatively well represented in MOZART-3, quantitatively, the trace gas profiles show some discrepancies. The main factor that determines the model's accuracy of chemical distribution in the UTLS region is the meteorological data: MOZART-3 driven by the ECMWF reanalysis winds has shown the best agreement with observational data[2,9].

Modelling data

The 3D CTM, MOZART-3, set-up is described in detail in the Methods section. The monthly averages, starting in January and finishing in December, representing the year 2006 are exploited in the CTM comparison with observational data.

Measurement data

The summary of the geographical distributions of the selected observational stations and regions applied for this analysis is presented in Supplementary Figure 4.

World Data Centre for Greenhouse Gases (WDCGG) is part of the Global Atmosphere Watch (GAW) program of the World Meteorological Organization (WMO) and is led by the Japanese Meteorological Agency. This observational network consists of stationary and mobile (aircraft, ship) stations, as well as ice core data being available. In this study, the stationary stations are chosen across the globe and the monitored CO and NO_2 values were employed for this study. The data were downloaded from the WDCGG website http://ds.data.jma.go.jp/gmd/wdcgg/cgibin/wdcgg/catalogue.cgi/.

World Ozone and Ultraviolet Radiation Data Centre (WOUDC) is another project of the Global Atmosphere Watch (GAW) program of the World Meteorological Organization (WMO) and is operated by the Meteorological Service of Canada. WOUDC provides the O_3 and ultraviolet

radiation observations, which are represented by more than 400 stations. The observations have been run for more than 50 years and they consist of total column ozone, the vertical profiles from ozonesondes, lidar measurements and the umkehr technique. For the purpose of this study, the ozonesondes data for selected ozone stations for the year 2006 were utilized. The daily profiles for selected months, January, April, July, October, were employed here. The data were downloaded from the WOUDC FTP server: ftp://woudc:woudc*@ftp.tor.ec.gc.ca/.

The Southern Hemisphere Additional Ozonesondes (SHADOZ) project was originated by NASA/Goddard Space Flight Centre and other U.S. and international investigators in 1998. The main aims are to provide the climatological profiles of tropical ozone in the equatorial zone and to validate and refine satellite remote sensing techniques for estimating tropical ozone estimations. Currently, the observational network consists of eleven stations that launch ozonesondes. For the purpose of this study, five stations that provide ozone profiles for the year 2006, were chosen. The daily profiles for the selected months, January, April, July, October, were employed here. The data were downloaded from the SHADOZ/Data Archive website http://croc.gsfc.nasa.gov/shadoz/.

The TOPSE campaign (Tropospheric Ozone Production about the Spring Equinox) was undertaken in spring 2000. This aircraft campaign covered the continental part of North America with a latitudinal range from 37°N to 90°N and from100 ft to 25000 ft in altitude. TOPSE measurements give a unique view of the spatial and temporal distribution of ozone and ozone precursors. The gridded climatologies and the regional profiles are provided; the latter were utilized in this study. The 3 regions used were defined in TOPSE campaign as follows: Boulder (37-47°N, 250-270°E), Churchill (47-65°N, 250-280°E) and Thule (65-90°N, 250-300°E). The O_3, NO_x, NO_y, CO, CH_4, PAN constituents were chosen for analysis. The data were downloaded from the website of the Atmospheric Chemistry Division at NCAR http://www.acd.ucar.edu/gctm/data/.

The LIS/OTD climatology datasets are a gridded climatology of total lightning flash rates, derived from two lightning detection sensors - the spaceborne Optical Transient Detector (OTD) on Orbview-1 and the Lightning Imaging Sensor (LIS) onboard the Tropical Rainfall Measuring Mission (TRMM) satellite. The LIS record makes the merged climatology most robust in the tropics and subtropics, while the high latitude data is entirely from OTD. Here we use the High-Resolution Monthly Climatology (HRMC) gridded product version 2.3[10].

The HRMC consists of long-term monthly mean flashes rate ($km^{-2}\ d^{-1}$) from OTD that has been collected from May 1995 to December 2000 and from LIS data that adds the years 1998-2014. The gridded satellite lightning datasets are available from the NASA's Global Hydrology and Climate Center at Marshall Space Flight Center, http://ghrc.msfc.nasa.gov.

Analysis

Time series of CO and NO_2 for the year 2006 (based on WDCGG data) and monthly O_3 profiles for the year 2006 (based on WOUDC and SHADOZ data) were analysed, along with O_3 and its precursors at Northern mid-latitudes and polar region during spring 2000 (based on TOPSE campaign data). The selected stations and regions are presented in Supplementary Figure 4.

In general, the magnitudes and temporal variations of NO_2 and CO are reproduced well by the model, despite some discrepancies. The distributions of NO_2 concentrations over Europe (represented by 6 ground stations) reveal good agreement with modelled volume mixing ratios (Supplementary Figure 5). In most cases, differences in the mean of NO_2 for the year 2006 do not exceed 15%. The exception is at stations Zoseni and Burgas, for which the MOZART-3 annual mean is overestimated by 43%, and underestimated by 34%, respectively. At Zoseni the differences are observed during winter months, whereas at Burgas the spring and autumn time causes discrepancies. This might originate from the anthropogenic emission input dataset compared with real localized emissions.

The CO observations (represented by 9 ground stations) are more spread over the globe. Again, the model and observational data are generally in good agreement and seasonal dependencies are reproduced well by MOZART-3 (Supplementary Figure 6). However, some tendencies are noticeable. The modelled mean of CO concentrations for the year 2006 at high and mid-northern latitudes are similar to the observed data and the differences are in the range of 2%. At the tropical northern station modelled values are underestimated by 13%, compared with the measurements. The MOZART-3 results show consistently larger CO concentrations at southern latitudes than the observations, with annual mean differences ranging from 14% at Casey Station, 17% at Cape Ferguson, to 28% at Cape Point.

The monthly O_3 profiles from 9 ozonesonde stations were compared with O_3 from MOZART-3 simulations, for the year 2006 (Supplementary Figures 7, 8, 9). The good accuracy in reproducing the vertical distribution of O_3 in the troposphere and stratosphere is shown for

mid- and high latitudes of both hemispheres. The correlation coefficients are above 0.82 for the domain 1000-10 hPa. However, the excessive vertical dispersion in the upper troposphere is observed among some of the MOZART-3 profiles. This is especially pronounced at the tropical tropopause represented by Paramaribo, Hilo, Ascension Island, Ha Noi, Kuala Lumpur. In the 250-150 hPa region, the modelled values reach their largest overestimations in April at the Paramaribo station, with its 45 ppbv (58%) O_3 difference. The larger O_3 production in the upper troposphere in MOZART-3 is an issue that was already pointed out by van Noije et al.[11] and Kinnison et al.[2]. The latter explains this through too strong Brewer-Dobson circulation in MOZART-3, which is one of the difficulties when the accurate representation of dynamical and transport processes in the UTLS needs to be modelled[12].

The TOPSE aircraft campaign is extremely useful as it gives the unique opportunity to validate the simulated O_3 and its precursors during the springtime in the mid- and high northern latitudes. The observational and modelled data for 7 altitudinal bands is shown in Supplementary Figure 10. The accuracy of simulated concentrations is generally good, and the correlation coefficients are usually high, greater than 0.9 for O_3, CH_4, CO and NO_y and 0.7 for NO_x and PAN. There is a certain pattern, which can be observed; that the oxidized nitrogen species (NO_x, NO_y, and PAN) are overestimated by MOZART-3 near the surface, which is observed especially in the Boulder region of the US. This discrepancy decreases with height when the distance from anthropogenic emissions is increasing. For example, the modelled NO_x at the surface is 7 times larger than observed concentrations, to become consistent within 10% one layer (1.5 km) higher (in the Churchill region). On the contrary, the CO and CH_4 concentrations are slightly underestimated in MOZART-3 in each region for each altitudinal band. However, the differences are relatively small and did not exceed 1.5% for CH_4 in each region and for CO 15% in Thule and Churchill and 25% in Boulder. Concentrations of O_3 are the best reproduced in Thule and Churchill (differences are within 10%) and relatively poorer accuracy is observed in the Boulder region (differences are within 25%). Most of the concentrations simulated by the model lie within 1-σ of the observational data (PAN near the surface is an exception).

The NO_x emission from lightning (LNO_x) modelled by MOZART-3 is compared with LIS/OTD climatology datasets[10]. There is a general agreement in the patterns of lighting NO_x distribution with some discrepancies in the regional size of the emissions (Supplementary Figure 11). LNO_x emissions are observed mainly in the Northern Hemisphere in summer and

the Southern Hemisphere in the northern hemisphere winter and it is observed both in modelled and measurement data. However, while in the tropics modeled LNO_x shows good agreement with satellite climatologies, there is a consistent underestimation of lightning emissions in MOZART-3 in mid-latitudes, especially during spring and autumn. MOZART-3 distributes LNO_x emissions vertically through the convective column according to observed profiles based on Pickering et al.[13]. If the more recent vertical profiles are applied[14] the zonal mean NO_x might increase by ~10 % in the middle troposphere and decrease by ~15 % in the tropical upper troposphere[15]. However, the effect on simulated O_3 would be rather small, at most a few percent[15,16]. When it comes to global flash rates and LIS/OTD satellite data, no instrument can determine precisely the number, length, peak current and energy of the flashes, and the lightning observations may miss some low-current flash events contributing to NO_x production[17]. Nevertheless, the total global emission of NO_x from lightning in MOZART-3, 4.7 Tg (N) yr^{-1}, is in agreement with the best estimates available in the literature, (5±3) Tg (N) yr^{-1} [17]. Despite the fact that the importance of the LNO_x emissions for upper-tropospheric chemistry and aircraft net NO_x estimates[18] are well known, LNO_x emissions remain highly uncertain[19, 20].

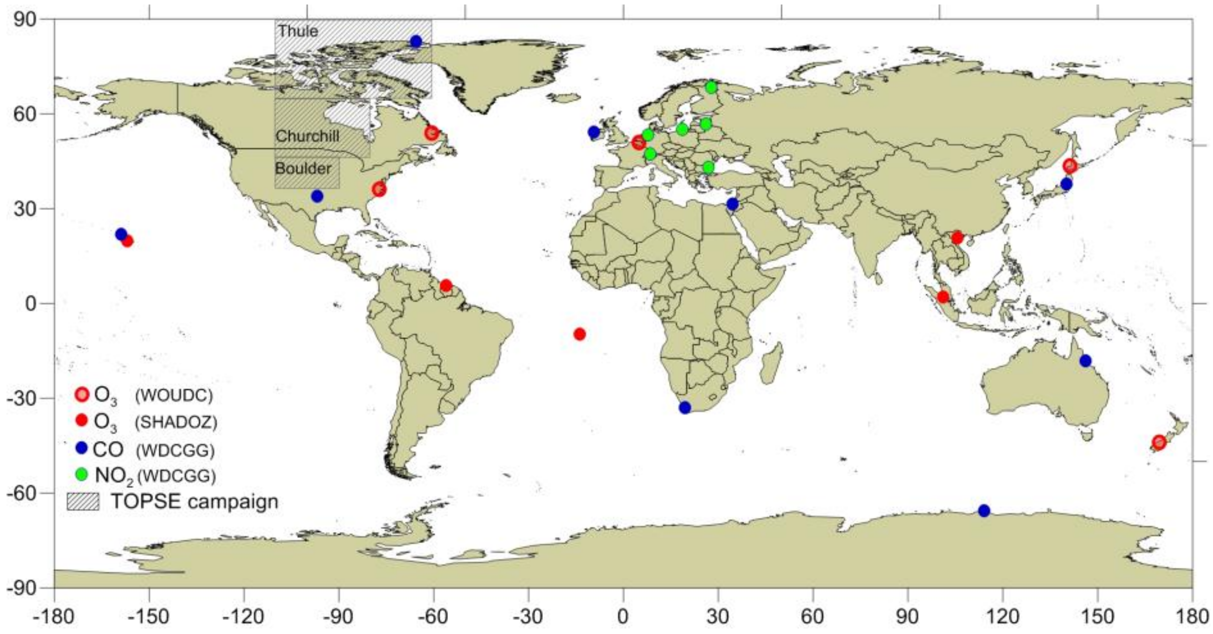

Supplementary Figure 4. Locations of WOUDC and SHADOZ ozonesonde stations, WDCGG stationary stations and geographical regions covered by aircraft TOPSE campaign.

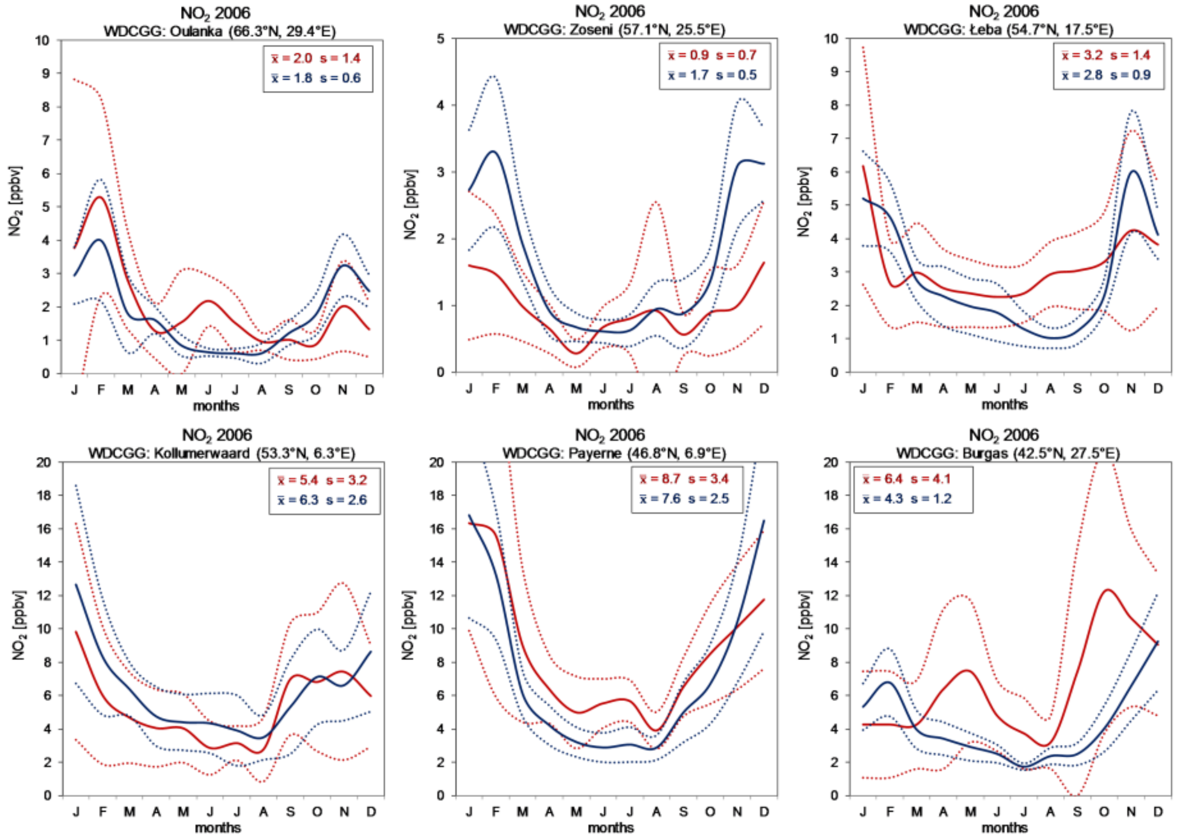

Supplementary Figure 5: Time series of NO_2 [ppbv] concentrations in 2006 from 6 WDCGG ground stations (red lines) and the corresponding time series simulated by MOZART-3 (blue lines). The dotted lines show the standard deviation range. WDCGG and MOZART-3 annual means and their standard deviations are provided for each location.

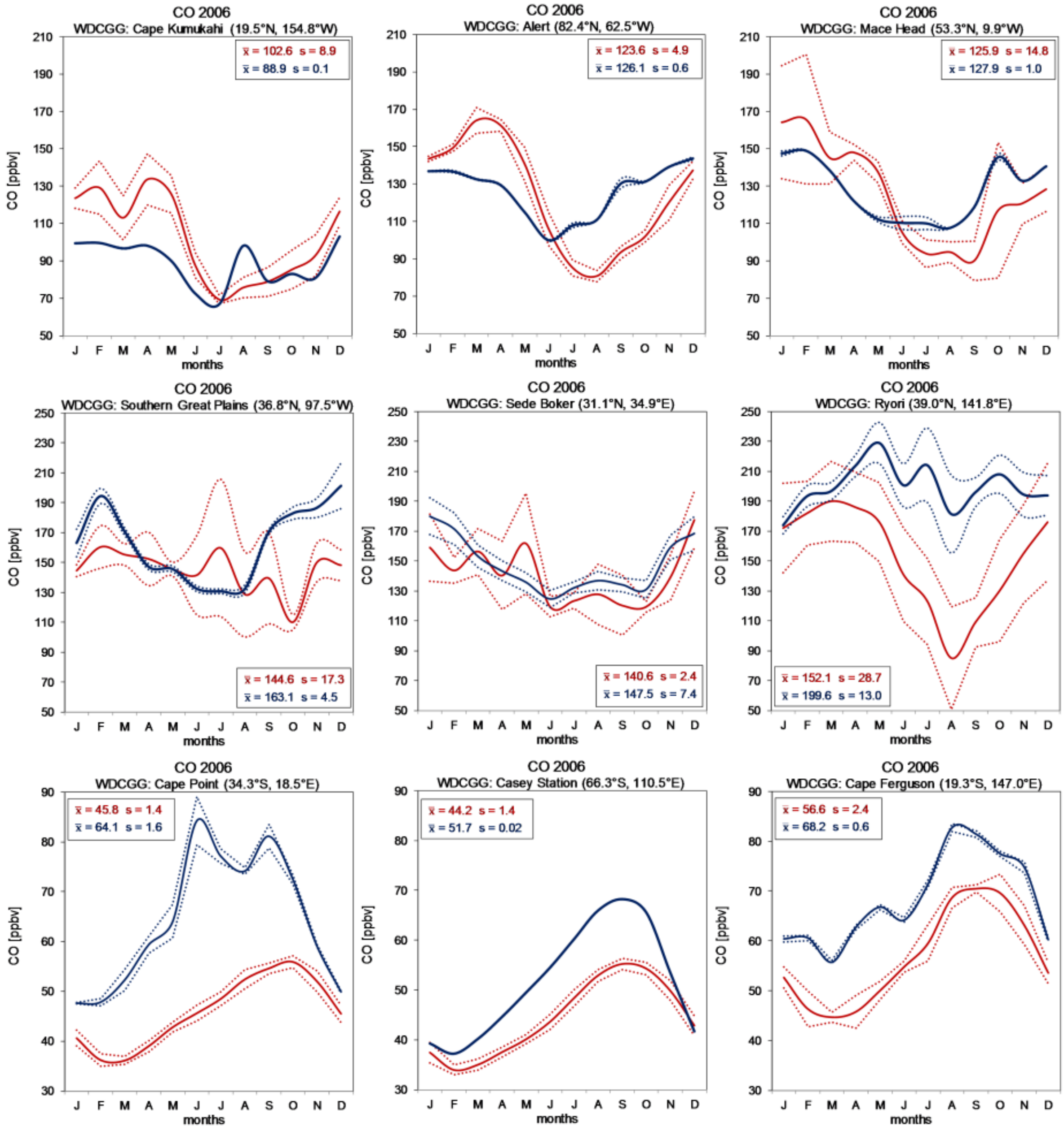

Supplementary Figure 6: Time series of CO [ppbv] concentrations in 2006 from 9 WDCGG ground stations (red lines) and the corresponding time series simulated by MOZART-3 (blue lines). The dotted lines show the standard deviation range. WDCGG and MOZART-3 annual means and their standard deviations are provided for each location.

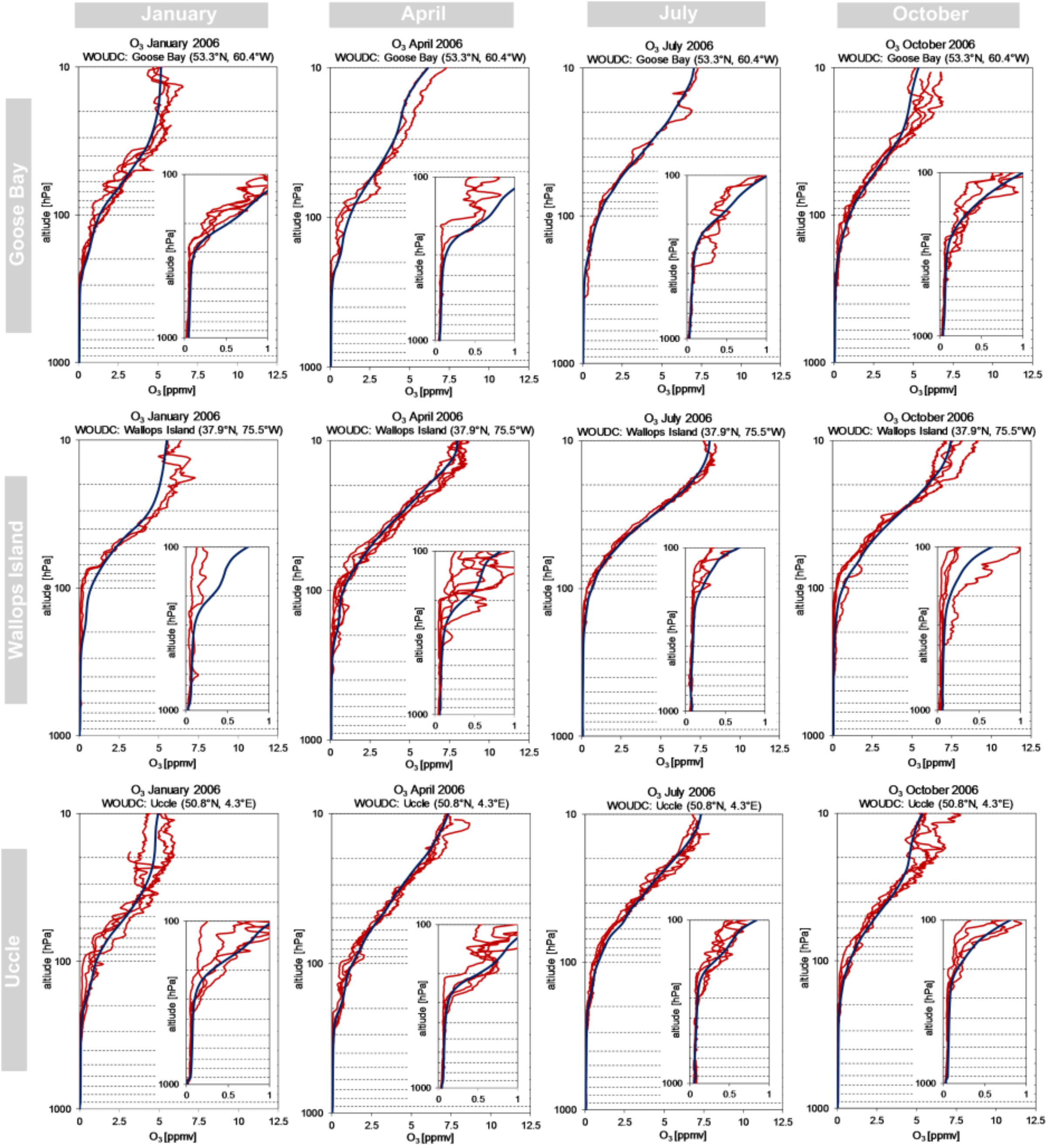

Supplementary Figure 7: Monthly mean vertical profiles of O_3 [ppmv] in 2006 measured at selected, 5 WOUDC and 4 SHADOZ, sonde stations (red line) and modelled by MOZART-3 (blue line).

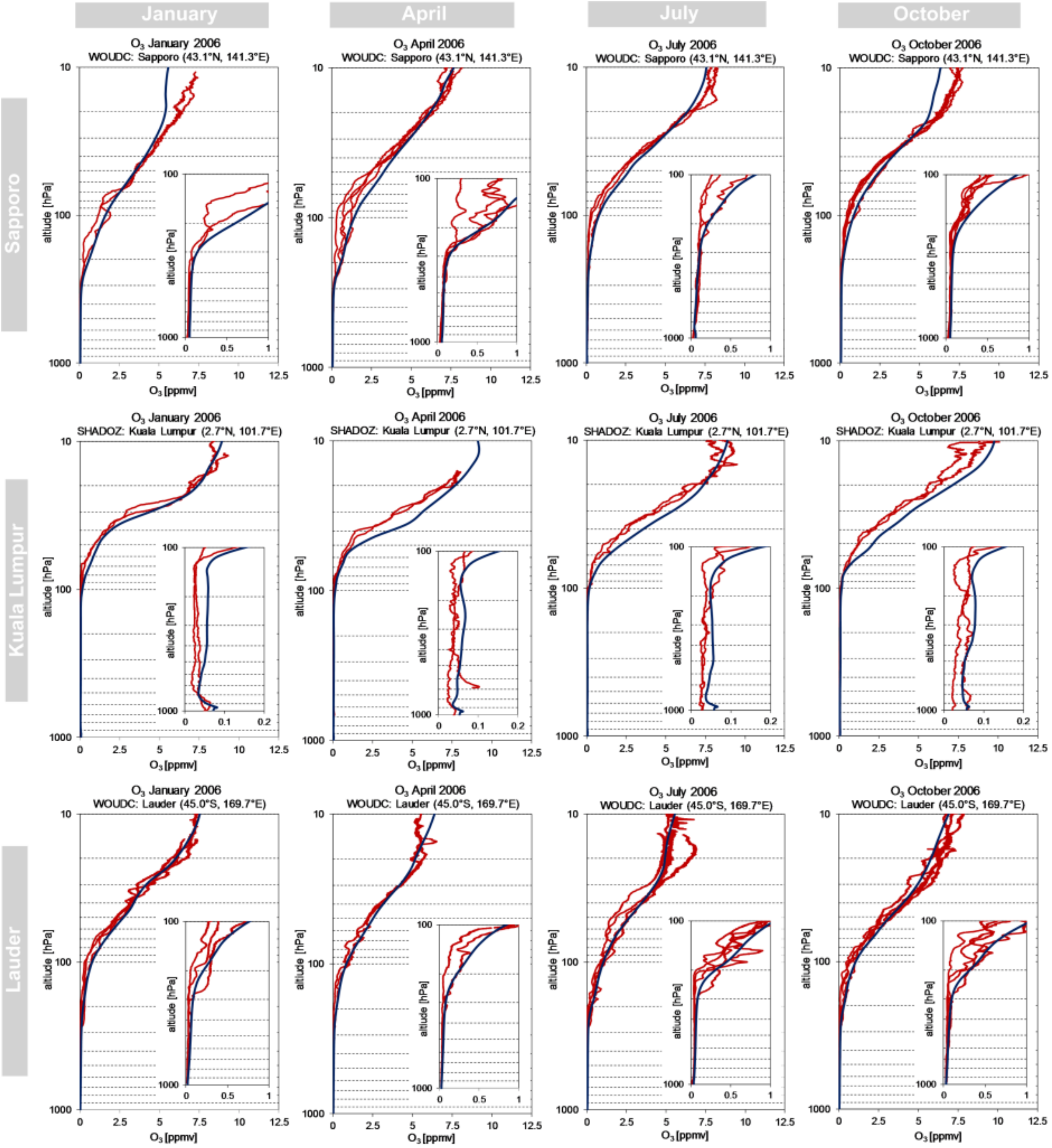

Supplementary Figure 8: Continuation of Supplementary Figure 7.

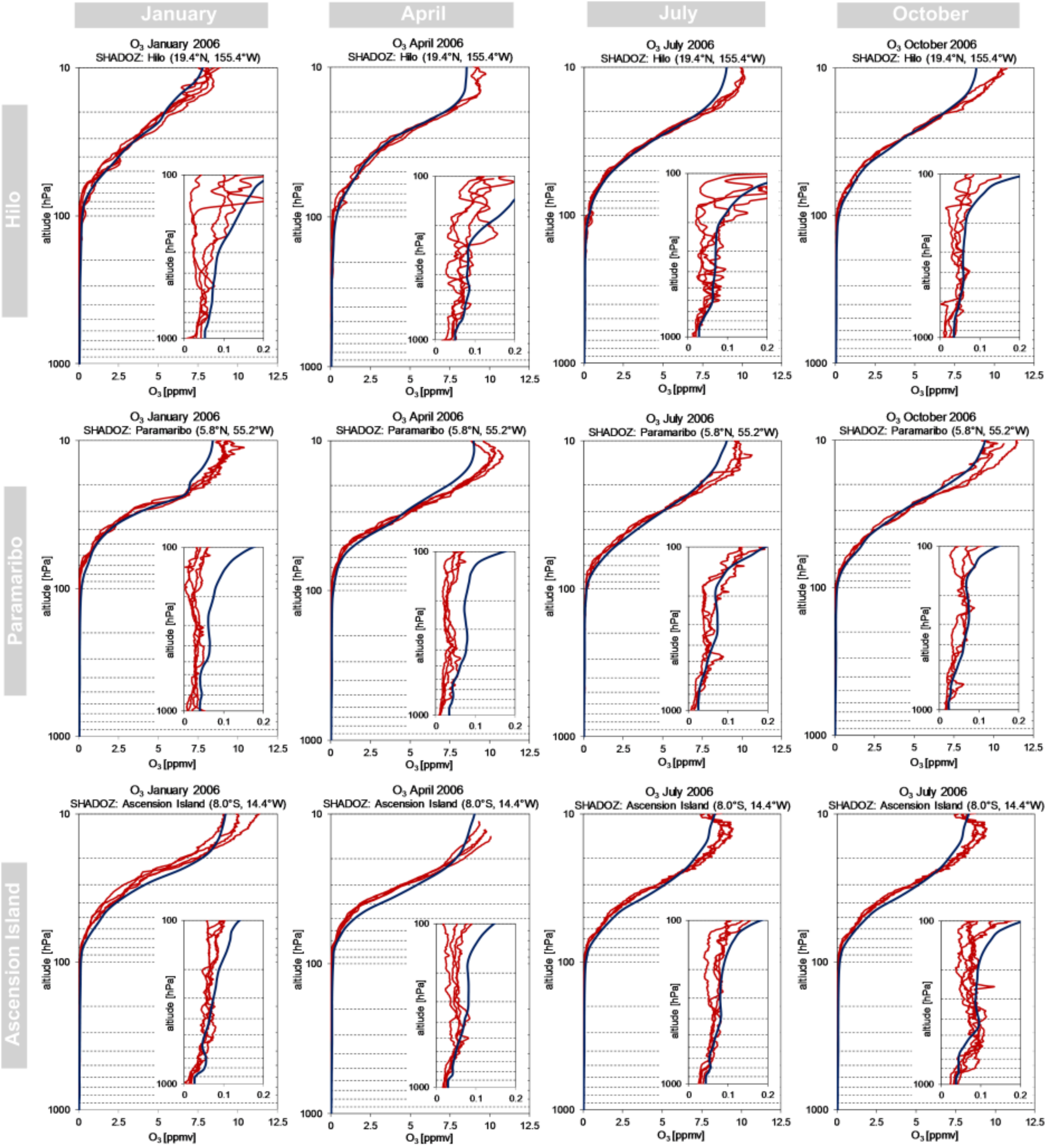

Supplementary Figure 9: Continuation of Supplementary Figure 7.

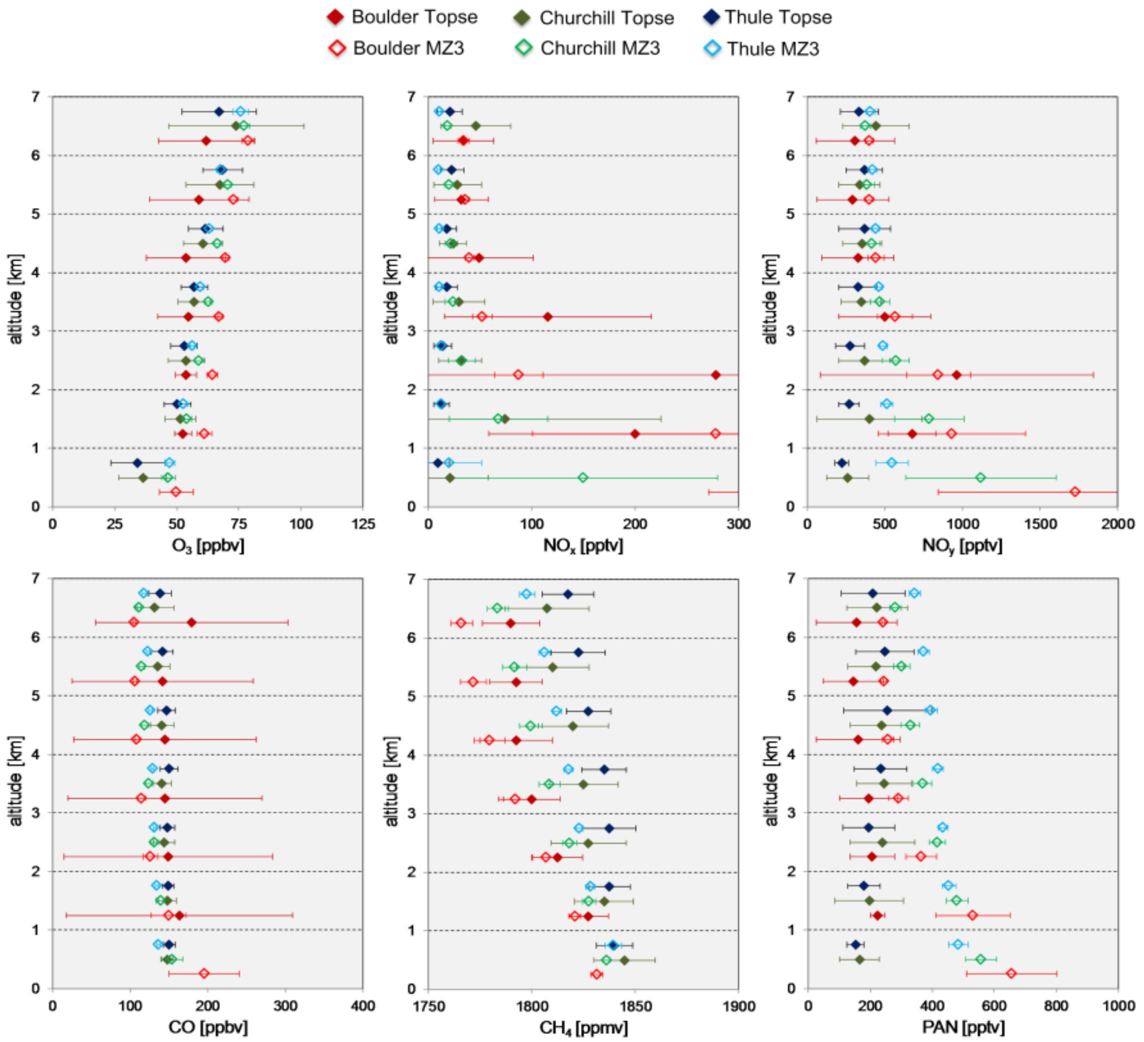

Supplementary Figure 10: Comparison of O_3, NO_x, NO_y, CO, CH_4, PAN concentrations between measurements taken during the TOPSE campaign and MOZART-3. The means (dots) and standard deviations (lines) constitute an average of 4 months (February, March, April, May) of year 2000.

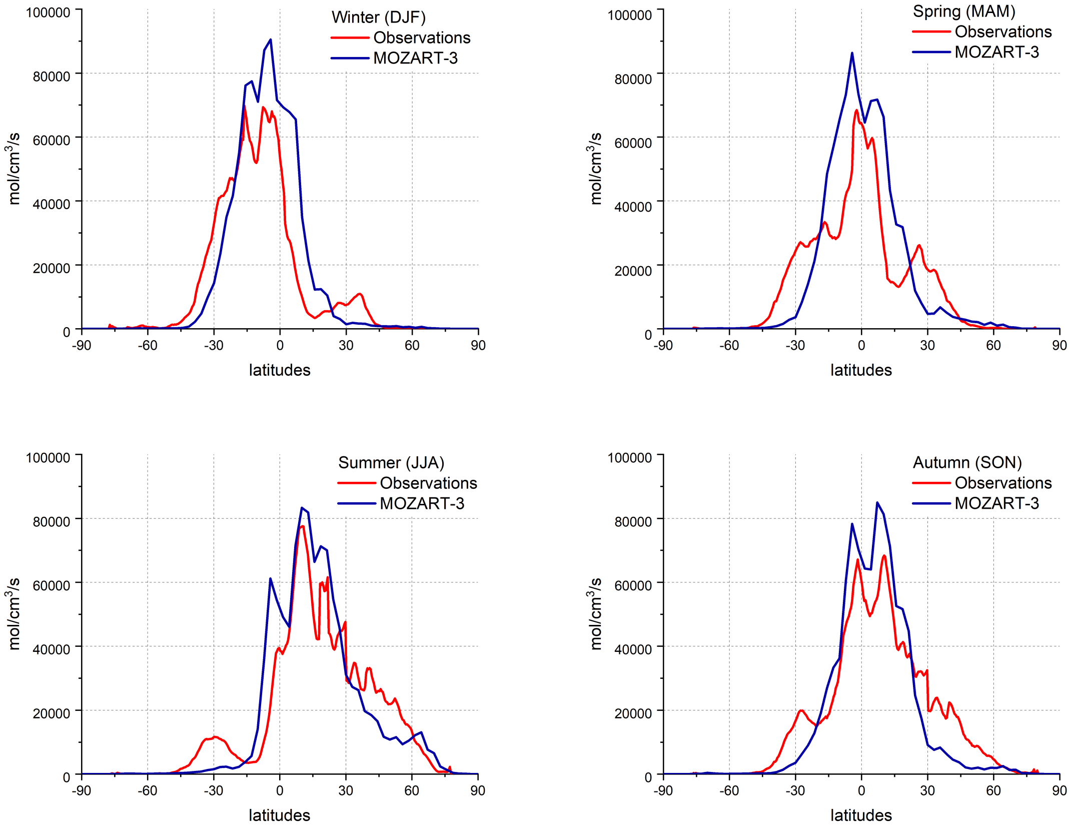

Supplementary Figure 11: Globally and seasonally averaged NO_x emissions from lightning as computed from MOZART-3 (blue) and the LIS/OTD High-Resolution Monthly Climatology data, version 2.3 (red). The yield of 250 mol N per flash[17] has been considered here. The gridded satellite lightning data is available at http://ghrc.msfc.nasa.gov.

Supplementary Note 2: The validity of the usage of MOZART-3, the 2-years simulation and the interannual meteorological variability in representing the aircraft O_3 response

The tool applied in this study is the model for ozone and related chemical tracers, version 3 (MOZART-3) and it is a 3D Chemistry Transport Model (CTM). CTMs are driven with fixed offline meteorology that does not incorporate any interactions and feedbacks between chemistry and climate/meteorology. MOZART-3 is a reliable tool in modelling the aircraft NO_x emissions[9] and is not an outlier among modelled estimates (Supplementary Figure 1). As Supplementary Figure 1 shows, Chemistry Climate Models (CCMs) have been also widely exploited. They are a modern generation of models embedded within the GCM environment and can be run in a variety of modes. Here, based on available studies, only two aircraft net NO_x estimates come from the CCMs with included feedback processes and coupled interactions, the rest have been run in an offline mode. An analysis of the distribution and the statistics of aircraft net NO_x RFs derived by CTM and CCMs has been made and is presented in Supplementary Figure 12. Both datasets are characterized by a normal distribution and the means are 4.93 and 4.11 mW m^{-2}/Tg(N) yr^{-1} for CTMs and offline CCMs, respectively. The difference between the median of these two datasets is greater than the mean, and is 2.35 mW m^{-2}/Tg(N) yr^{-1}. Most of the net NO_x RFs can be found between 8 and 10 mW m^{-2}/Tg(N) yr^{-1} for both CTMs and offline CCMs. A Welch's t-test has been also performed and showed that the null hypothesis cannot be rejected ($t(26) = 0.524$, $p = 0.605$), which means there is no statistically significant difference between aircraft net NO_x RFs derived by CTMs and offline CCMs.

The calculations of O_3 change and CH_4 lifetime change, along with RFs, covers the surface-1 hPa domain. Since the experiments are performed for 2 years, the magnitude of the aviation stratospheric O_3 response is not fully representative (Supplementary Figure 12). Stratospheric transport timescales are of several years[21] and indeed an increased aircraft O_3 response in the stratosphere is still observed in the 6th year of simulation, with magnitudes much more enhanced than for 2 years of aircraft perturbation runs (Supplementary Table 2). The annual average stratospheric (100-1 hPa) O_3 column change in the 2nd year is positive (with July showing the most negative peak -0.0002 DU), whilst the 6th year shows negative O_3 change through all year (with greatest July depletion -0.0137 DU). Despite these significant differences in the stratospheric response to aviation NO_x emissions between the 2nd and 6th year, the total O_3 change is not as much affected, as most of the mass of aircraft O_3 is

concentrated in the UTLS region. The difference in O_3 column change between the 2nd and 6th year is 5.1%, the differences in the resultant O_3 RF is -0.6%. Thus, the O_3 changes presented in this work can be treated as reliable.

The impact of the interannual meteorological variability on aircraft O_3 response has been also explored (Supplementary Figure 13). The MOZART-3 has been driven by the fields from the European Centre for Medium Range Weather Forecast (ECMWF), 6-h reanalysis ERA-Interim data[22] for four different years: 2004, 2005, 2006 and 2007. It is observed that the annual pattern of aircraft O_3 is consistent for each specific year of the meteorological condition and the monthly O_3 changes from aircraft NO_x emissions are within 5% of each other. The global annual means of aircraft O_3 change are calculated to be 0.513 DU, 0.514 DU, 0.508 DU, and 0.515 DU for the years 2004, 2005, 2006 and 2007 respectively, where the aircraft O_3 in the year 2006 being the smallest among the investigated years, but still in agreement, within 2% with other meteorological conditions. The uncertainty in aircraft O_3 response arising from interannual meteorological variability is estimated here to be a rather of a low significance.

Supplementary Table 2: The global and annual mean O_3 column change (in DU) and RF response due to aircraft O_3 for consecutive years of MOZART-3 simulations.

Year	aircraft O_3 [DU]			aircraft O_3 RF [mW m^{-2}]
	surf - 100 hPa	100 - 1 hPa	total	Net
2nd	0.516	0.002	0.518	13.4
5th	0.503	-0.010	0.493	-
6th	0.503	-0.011	0.492	13.5

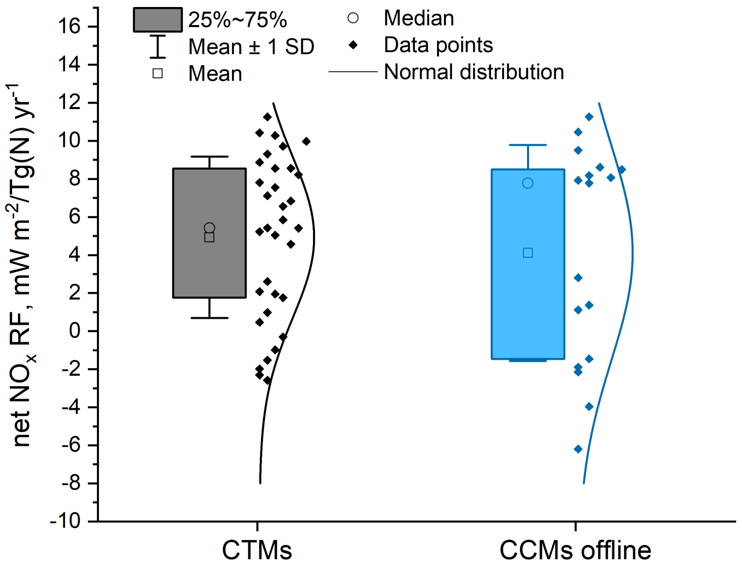

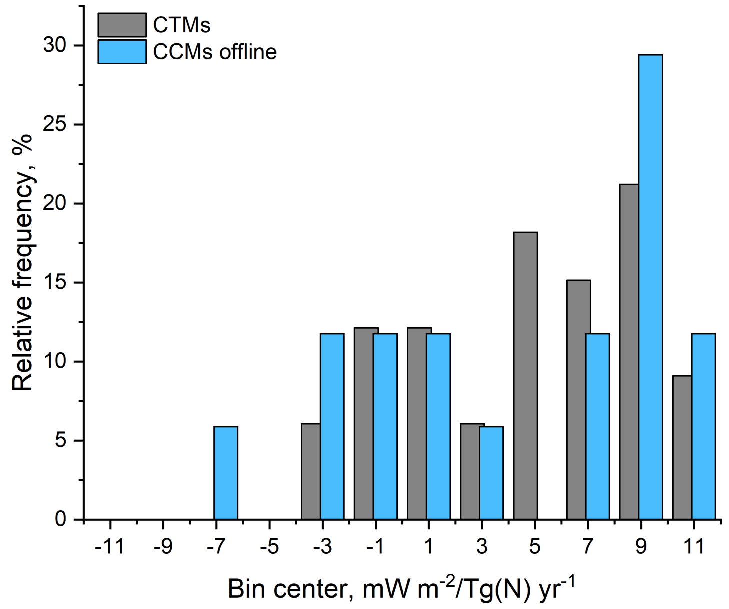

Supplementary Figure 12: The results of the global and annual net NO_x RFs from aircraft studies as presented in Supplementary Figure 1. The analysis covers a wide range of global atmospheric chemistry/climate models: grey – CTMs, blue – CCMs offline, run in CTM mode (green – CCM online has been excluded due to too few estimates). The upper panel presents a descriptive statistic that summarizes the net NO_x RF derived by CTMs (grey) and offline CCMs (blue). The bottom panel shows the characteristic of the distribution of aircraft net NO_x RF derived by CTMs (grey) and offline CCMs (blue).

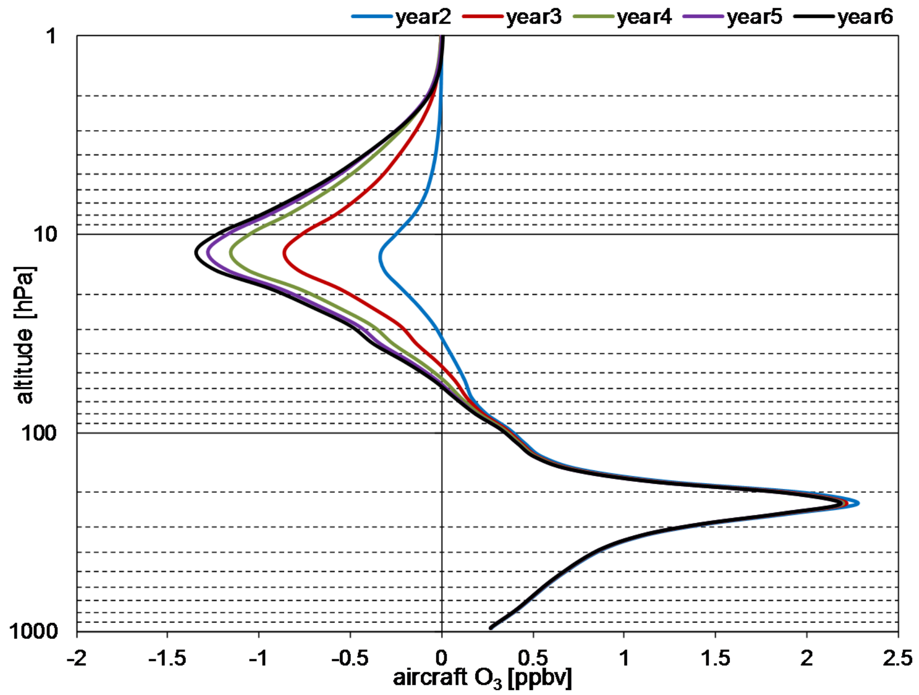

Supplementary Figure 13: The globally and annually averaged vertical distributions of aircraft perturbations of O_3 concentrations for consecutive years of simulations.

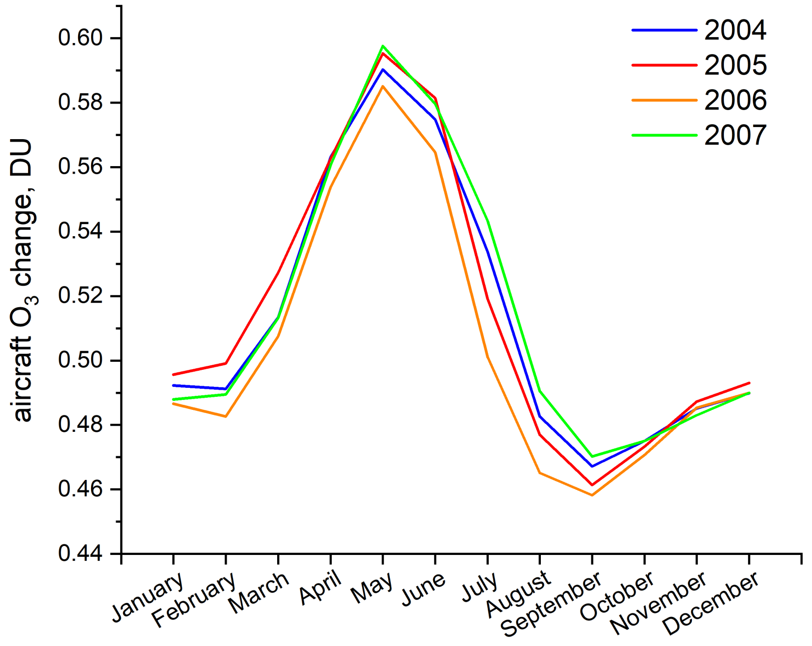

Supplementary Figure 14: The globally averaged monthly distributions of aircraft O_3 perturbations as modelled by MOZART-3 driven by the meteorological fields from the European Centre for Medium Range Weather Forecast (ECMWF), 6-h reanalysis ERA-Interim data for the years 2004, 2005, 2006 and 2007.

Supplementary References:

1. Lee, D. S. et al. The contribution of global aviation to anthropogenic climate forcing for 2000 to 2018. *Atmos. Environ.* **244**, 117834 (2021).
2. Kinnison, D. E. et al. Sensitivity of chemical tracers to meteorological parameters in the MOZART-3 chemical transport model. *J. Geophys. Res.* **112**, D20302 (2007).
3. Flemming, J. et al. Forecasts and assimilation experiments of the Antarctic ozone hole 2008. *Atmos. Chem. Phys.* **11**, 1961-1977 (2011).
4. Sassi, F., Kinnison, D. E., Boville, B. A., Garcia R. R. & Roble R. Effect of El Nino – Southern Oscillation on the dynamical, thermal, and chemical structure of the middle atmosphere. *J. Geophys. Res.* **109**, D17108 (2004).
5. Liu, Y. et al. Atmospheric tracers during the 2003−2004 stratospheric warming event and impact of ozone intrusions in the troposphere. *Atmos. Chem. Phys.* **9**, 2157−2170 (2009).
6. Pan, L. L. et al. A set of diagnostics for evaluating chemistry climate models in the extratropical tropopause region. *J. Geophys. Res.* **112**, D09316 (2007).
7. Gettelman, A., Kinnison, D. E., Brasseur, G. & Dunkerton T. Impact of monsoon circulations on the upper troposphere and lower stratosphere. *J. Geophys. Res.* **109**, D22101 (2004).
8. Park, M., Randel, W. R., Kinnison, D. E., Garcia, R. R. & Choi W. Seasonal variations of methane, water vapor, ozone, and nitrogen dioxide near the tropopause: Satellite observations and model simulations. *J. Geophys. Res.* **109**, D03302 (2004).
9. Freeman, S., Lee, D. S., Lim, L. L., Skowron, A. & De Leoń, R. R. Trading off aircraft fuel burn and NO_x emissions for optimal climate policy. *Environ. Sci. Technol.* **52**, 2498−2505 (2018).
10. Cecil, D. J., Buechler, D. E. & Blakeslee, R. J. Gridded lightning climatology from TRMM-LIS and OTD: Dataset description. *Atmos. Res.* **135-136**, 404-414 (2014).
11. van Noije, T. P. C. et al. Multi-model ensemble simulations of tropospheric NO_2 compared with gome retrievals for the year 2000. *Atmos. Chem. Phys.* **6**, 2943-2979 (2006).
12. Schoeberl, M. R., Douglass, A. R., Zhu, Z. & Pawson S. A comparison of the lower stratospheric age spectra derived from a general circulation model and two data assimilation systems, *J. Geophys. Res.* **108** (D3), 4113 (2003).
13. Pickering, K. E., Wang, Y., Tao, W.-K., Price, C. & Müeller, J.-F. Vertical distributions of lightning NOx for use in regional and global chemical transport models. *J. Geophys. Res*. **103**, 31203-31216 (1998).
14. Ott, L. E. et al. Production of lightning NO_x and its vertical distribution calculated from three-dimensional cloud-scale chemical transport model simulations. *J. Geophys. Res.* **115**, D04301 (2010).
15. Søvde, O. A. et al. The chemical transport model Oslo CTM3. *Geosci. Model Dev.* **5**, 1441-1469 (2012).
16. Murray, L. T., Jacob, D. J., Logan, J. A., Hudman, R. C. & Koshak, J. Optimized regional and interannual variability of lightning in a global chemical transport model constrained by LIS/OTD satelite data. *J. Geophys. Res.* **117**, D20307 (2012).
17. Schumann, U. & Huntrieser, H. The global lightning-induced nitrogen oxides source. *Atmos. Chem. Phys.*, **7**, 3823–3907 (2007).
18. Khodayari, A., Vitt, F., Phoenix, D. & Wuebbles, D. J. et al. The impact of NO_x emissions from lightning on the production of aviation-induced ozone. *Atmos. Environ.* **187**, 410-416 (2018).
19. Gressent, A. et al. Modeling lightning-NO_x chemistry on a sub-grid scale in a global chemical transport model. *Atmos. Chem. Phys.* **16**, 5867-5889 (2016).
20. Gordillo-Vazquez, F. J., Pérez-Invernón, F. J., Huntrieser, H., & Smith, A. K. Comparison of six lightning parameterizations in CAM5 and the impact on global atmospheric chemistry. *Earth Space Sci.e* **6**, 2317-2346 (2019).
21. Stiller, G. P. et al. Observed temporal evolution of global mean age of stratospheric air for the 2002 to 2010 period. *Atmos. Chem. Phys.* **12**, 3311-3331 (2012).
22. Dee, D. P. et al. The ERA-interim reanalysis: configuration and performance of the data assimilation system. *Q. J. R. Meteorol. Soc.* **137**, 553-597 (2011).
