# Supplementary material for: Greater fuel efficiency is potentially preferable to reducing NOx emissions for aviation’s climate impacts
Source: Nat Commun. 2021 Jan 25;12:564. doi: 10.1038/s41467-020-20771-3 (PMC7835228; doi:10.1038/s41467-020-20771-3)
Supplement: Supplementary file 3 — Reporting Summary [file 41467_2020_20771_MOESM3_ESM.pdf]

nature research

Corresponding author(s): Agnieszka Skowron

Last updated by author(s): Dec 9, 2020

Reporting Summary

Nature Research wishes to improve the reproducibility of the work that we publish. This form provides structure for consistency and transparency in reporting. For further information on Nature Research policies, see Authors & Referees and the Editorial Policy Checklist.

Statistics

For all statistical analyses, confirm that the following items are present in the figure legend, table legend, main text, or Methods section.

n/a	Confirmed	
☐	☒	The exact sample size (*n*) for each experimental group/condition, given as a discrete number and unit of measurement
☒	☐	A statement on whether measurements were taken from distinct samples or whether the same sample was measured repeatedly
☐	☒	The statistical test(s) used AND whether they are one- or two-sided<br>*Only common tests should be described solely by name; describe more complex techniques in the Methods section.*
☒	☐	A description of all covariates tested
☒	☐	A description of any assumptions or corrections, such as tests of normality and adjustment for multiple comparisons
☐	☒	A full description of the statistical parameters including central tendency (e.g. means) or other basic estimates (e.g. regression coefficient) AND variation (e.g. standard deviation) or associated estimates of uncertainty (e.g. confidence intervals)
☐	☒	For null hypothesis testing, the test statistic (e.g. *F*, *t*, *r*) with confidence intervals, effect sizes, degrees of freedom and *P* value noted<br>*Give P values as exact values whenever suitable.*
☒	☐	For Bayesian analysis, information on the choice of priors and Markov chain Monte Carlo settings
☒	☐	For hierarchical and complex designs, identification of the appropriate level for tests and full reporting of outcomes
☒	☐	Estimates of effect sizes (e.g. Cohen's *d*, Pearson's *r*), indicating how they were calculated

Our web collection on statistics for biologists contains articles on many of the points above.

Software and code

Policy information about availability of computer code

Data collection	3D CTM, MOZART-3 run on CRAY Supercomputer
Data analysis	IDL 8.5.1, Origin 2019 (9.6), Surfer 7

For manuscripts utilizing custom algorithms or software that are central to the research but not yet described in published literature, software must be made available to editors/reviewers. We strongly encourage code deposition in a community repository (e.g. GitHub). See the Nature Research guidelines for submitting code & software for further information.

Data

Policy information about availability of data

All manuscripts must include a data availability statement. This statement should provide the following information, where applicable:

- Accession codes, unique identifiers, or web links for publicly available datasets
- A list of figures that have associated raw data
- A description of any restrictions on data availability

All data discussed in the manuscript and Supplementary Information (SI) are presented in Source Data. All data generated for this study (2006 and 2050 CTM and RTM simulations) are available on request from the corresponding author.

Field-specific reporting

Please select the one below that is the best fit for your research. If you are not sure, read the appropriate sections before making your selection.

☐ Life sciences ☐ Behavioural & social sciences ☒ Ecological, evolutionary & environmental sciences

October 2018

Ecological, evolutionary & environmental sciences study design

All studies must disclose on these points even when the disclosure is negative.

Study description	Using a 3D CTM, MOZART-3, the changes in tropospheric composition and the net RF from aviation NOx emissions for 30% reductions in the present-day O3 precursor emissions and for a future range of scenarios were examined.
Research sample	The transport of chemical compounds is driven by the meteorological fields from the ECMWF, 6-h reanalysis EAR-Interim data for the year 2006. The aviation NOx emissions for the years 2006 and 2050 were determined based on the REACT4C base case dataset (CAEP/8 movements) and ICAO-CAEP aviation emission projections, respectively. The present-day anthropogenic and biomass burning emissions were taken from IPCC TAR. The 2050 gridded surface emissions (anthropogenic and biomass burning) constitute the three Representative Concentration Pathways (RCP): RCP 2.6, RCP 4.5, RCP 8.5.
Sampling strategy	The monthly averages from MOZART-3 were exploited in the analysis.
Data collection	The MOZART-3 output has been directly saved on ssh askowron@cray.cate.mmu.ac.uk.
Timing and spatial scale	The model configuration used in this study includes a horizontal resolution of T42 (~2.8 x 2.8) and 60 hybrid layers, from the surface to 0.1 hPa. The runs have been performed for present-day (2006) and future (2050) conditions. Each experimental case consists of two years run.
Data exclusions	The first year of these two years simulations has been treated as a spin-up run and was excluded from the analysis.
Reproducibility	The sensitivity experiments have been performed and confirmed that e.g., running the model twice with exactly the same settings results in exactly the same output. The results of various sensitivity simulations have been presented in SI.
Randomization	Not applicable: data and analysis was based on atmospheric chemical modelling, using documented chemical kinetics and no data collection of samples was involved.
Blinding	Not applicable: no samples were collected, no data from dose-response functions were collected,

Did the study involve field work? ☐ Yes ☒ No

Reporting for specific materials, systems and methods

We require information from authors about some types of materials, experimental systems and methods used in many studies. Here, indicate whether each material, system or method listed is relevant to your study. If you are not sure if a list item applies to your research, read the appropriate section before selecting a response.

Materials & experimental systems

n/a	Involved in the study	
☒	☐	Antibodies
☒	☐	Eukaryotic cell lines
☒	☐	Palaeontology
☒	☐	Animals and other organisms
☒	☐	Human research participants
☒	☐	Clinical data

Methods

n/a	Involved in the study	
☒	☐	ChIP-seq
☒	☐	Flow cytometry
☒	☐	MRI-based neuroimaging
